# New faunistic data on Diptera (Hexapoda, Insecta) from the Ziarat *Juniperus* forest ecosystem (Pakistan)

**DOI:** 10.3897/BDJ.12.e114414

**Published:** 2024-03-25

**Authors:** Qaiser Khan, Asmathullah Kakar, Kashif Kamran

**Affiliations:** 1 Department of Zoology, University of Balochistan, Quetta, Pakistan Department of Zoology, University of Balochistan Quetta Pakistan

**Keywords:** Diptera, *Juniperus* forest, arcode index number, DNA barcode, cryptic species

## Abstract

**Background:**

This study presents the first faunistic record and DNA barcoding for some Diptera species recorded from the *Juniperus* forest ecosystem of Balochistan, Pakistan. DNA barcoding was used to explore species diversity of Dipterans and collections carried out using a Malaise trap between December 2018 to December 2019. This process involved sequencing the 658 bp Cytochrome Oxidase I (COI) gene.

**New information:**

Amongst the collected Diptera specimens, nine families were identified, representing 13 genera. These species include *Atherigonasoccata* (Rondani, 1871), *Atherigonavaria* (Schiner, 1868), *Chironomusdorsalis* (Meigen, 1818), *Eupeodescorollae* (Linnaeus, 1758), *Eristalistenax* (Linnaeus,1758), *Goniaornata* (Meigen, 1826), *Luciliasericata* (Meigen, 1826), *Paragusquadrifasciatus* (Linnaeus, 1758), *Polleniarudis* (Fabricius, 1794), *Raviniapernix* (Thompson, 1869), *Sarcophagadux* (Thompson, 1869), *Trupaneaamoena* (Schiner, 1868) and *Wohlfahrtiabella* (Linnaeus, 1758). The families Syrphidae and Sarcophagidae exhibited the highest representation, each comprising three genera and three species. They were followed by the family Muscidae, which had a single genus and two species. Anthomyiidae, Chironomidae, Calliphoridae, Polleniidae, Tachinidae and Tephritidae were represented by only one genus and one species. A nique Barcode Index Number (BIN) was allotted to Tachinidae (specie i.e *Goniaornata*). The results indicated that barcoding through cytochrome oxidase I is an effective approach for the accurate identification and genetic studies of Diptera species. This discovery highlights the significant diversity of this insect order in study region. Furthermore, a comprehensive list of other Diptera species remains elusive because of difficulties in distinguishing them, based on morphology and a lack of professional entomological knowledge.

## Introduction

Ziarat is a District in Pakistan that comprises green lush vista of *Juniperousexcelsa* (M.Bieb, 1798) trees with a total area of 3670 km^2^. It lies in the Province of Balochistan (outhwest Pakistan) and has an average altitude over 2000 m a.s.l. (Kamran et al. 2022). The area is mostly surrounded by high mountain ranges and hosts the world second largest and oldest *Juniperus* forest, with an average plant age over 1500 years (Jallat et al. 2021). Ziarat District and the *Juniperus* ecosystem in particular, is characteried by a dry climate, cool summers and cold winters, with precipitation and snow concentrat between December and March (Kamran et al. 2022). Due to its uniqueness, this area and its forest have been declared a ‘Ziarat Juniper Forest Biosphere Reserve’ by the United Nations Educational, Scientific and Cultural Organiation (UNESCO 2016). Juniper forest is considered part of the biological heritage of Pakistan (Jallat et al. 2021, Sarangzai et al. 2012). The Ziarat Juniper ecosystem is the habitat of important endangered species of wild animals, such as *Caprafalconerijerdoni* (Wagner, 1839), *Ovisvigneiblanfordi* (Hume, 1877), *Ursusthibetanusformosanus* (Swinhoe, 1864), *Canislupus* (Linnaeus, 1758), *Ellobiusfuscocapillus* (Blyth, 1843) (Ghalib et al. 2007) and also several others migratory species (Ghalib et al. 2019). This ecosystem hosts also several medically important plants, such as *Berberisbalochistanica* (*Uddin et al. 2021*), wild almond and *Ephedra* species etc. (Sarangzai et al. 2013). Pakistan encompasses four distinct biomes (Afsar and Bano 2013), all potentially hosting a high diversity in life forms. However, still little is known about the true diversity of invertebrate fauna, insects in particular and some of these biomes remain poorly investigated (Ashfaq et al. 2022). This knowledge gap is particularly marked for the Ziarat Juniper ecosystem, where the few known species are those of phytosanitary interest, such as pests on *Juniperus* and cultivated plants (Chaudhry 1979).

Specifically referring to Diptera, the fauna of Pakistan remains largely understudied due to the chronic scarcity of taxonomists (Scheffers et al. 2012, Dinerstein et al. 2017) and still much remains to be done to fill the knowledge gap, both with regard to the species presence and their distribution. In recent times, some contributions have started to help in bridging the gap providing important data for the outhern region of Punjab Province (Bashir et al. 2019, Hassan et al. 2017a), Punjab and Khayber Pakhtunkhua (Ashfaq et al. 2022), Azad Jammu & Kashmir (Hassan et al. 2017, Hassan et al. 2021) and Gilgit-Baltistan territories (Fatima and Yang 2022) and Sindh, where Syrphidae remains the most-studied family (e.g. Ansari and Memon (2017), Kanher (2022)).

The availability of reliable data regarding Diptera diversity in the Juniper ecosystem of Ziarat remains limited since this region has yet to be thoroughly investigated and only a few scattered data exist (e.g. Chaudhary and Aslam (2005), Javaid et al. (2006)). The primary objective of faunistic studies are to document and record medical and economic important species of insects (Ježek et al. 2021, Shehzad et al. 2017). These studies are crucial for assessing the abundance of species found within a specific geographical area and monitoring the long-term shifts in population trends and diversity (Ejsmont-Karabin 2019, Shamna et al. 2023). This has become particularly important to protected areas like Juniperus Ziarat, which hold immense biodiversity of significance, but has not received sufficient research attention for certain insect groups. Given the significance of the study area, the aim of this contribution is to increase reliable information on Diptera diversity of the area, providing standardised and geo-referenced occurrences and DNA barcodes (where possible) in order to provide a foundation for future studies.

## Materials and methods

### Material recollection and sequencing

**Figure 1.**
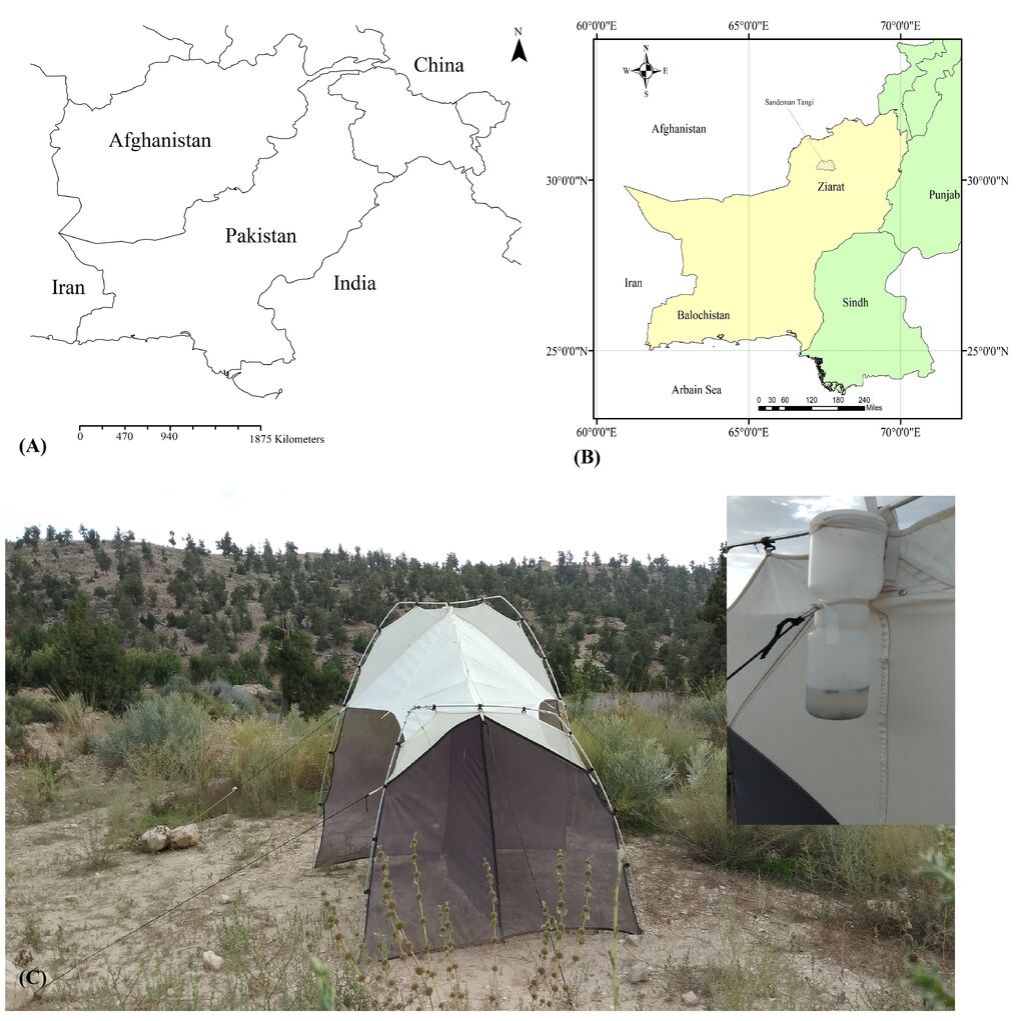
Sample collection site. **A** Map of Pakistan; **B** Ziarat District; **C** Malaise trap having insect collecting bottle installed at Sundaman Tangi.

A single malaise trap was installed at Sandamen Tangi (30^ᵒ^24'00.5" N: 67^ᵒ^43'36.5"E, 2450 m), (2450 m a.s.l.), a forested area located in the north of Ziarat City (Fig. 1). GPS coordinates of the sampling point were recorded using a Garmin eTrex 10 GSP device. The trap was activated on December 2018 and controlled at regular intervals of one week until the end of December 2019. During each visit, insects from the trap were transferred into Whirl-Pak bag containing 95% ethanol, labelled properly and susequentlly stored in a refrigerator at -20°C until processing. Diptera specimens were hand-sorted from alaise samples and morphologically identified to species level using monographs and identification keys (Alexander and Alexander 1965, Prabhakar et al. 2012, Sugiama 1989, Agarwal and Sueyoshi 2005, Chanthy et al. 2010, O’Hara et al. 2021, Yan et al. 2021, Mengual et al. 2020, Hassan et al. 2017a). For each species recorded, voucher specimens were dry-mounted and subsequently stored at the Entomology Laboratory of the Department of Zoology, University of Balochistan (Quetta, Pakistan). Voucher specimens of each species were submitted to Canadian Centre for DNA Barcoding (CCDB) (http://ccdb.ca/resources.php) for species identification through DNA sequencing following the specified barcoding procedures (deWaard et al. 2019a, deWaard et al. 2019b) and curated at the Centre for Biodiversity Genomics (https://biodiversitygenomics.net/), University of Guelph Canada. All the specimens were treated and analysed as per the protocol in the Biodiversity of Ontario Guelph University, Canada (Ivanova et al. 2006). The sequences along with BINs and other related taxonomic information were recorded on BOLD (Ratnasingham and Hebert 2007) and deposited in NCBI also (OR685682- OR685699). Then, these sequences were used for conducting comparative analysis and confirming identifications by lasting at NCBI. Under each species treated in the paper, we provided a direct link to the original sequence used for identification and stored in GenBank (NCBI).

## Taxon treatments

### Adia cinerella

(Fallén, 1825)

8E5B7CBB-BB98-50E5-9689-08FB75DBF41F

OR685682

OR685683

#### Materials

- **Type status:** Other material. **Occurrence:** recordedBy: Qaiser Khan; individualCount: 1; occurrenceID: 653C1014-DB51-5298-B513-9F4581D2EA8D; **Taxon:** scientificName: Adiacinerella (Fallén, 1825); order: Diptera; family: Anthomyiidae; genus: Adia; specificEpithet: cinerella; scientificNameAuthorship: (Fallén, 1825); **Location:** country: Pakistan; countryCode: PK; stateProvince: Balochistan; county: Ziatar District; municipality: Ziarat; decimalLatitude: 30.400139; decimalLongitude: 67.726806; geodeticDatum: WGS84; **Identification:** identifiedBy: Qaiser Khan; **Event:** eventDate: 2019-07-15
- **Type status:** Other material. **Occurrence:** recordedBy: Qaiser Khan; individualCount: 2; occurrenceID: BD07D5D1-E186-5A56-AFF0-EDEF4351AF89; **Taxon:** scientificName: Adiacinerella (Fallén, 1825); order: Diptera; family: Anthomyiidae; genus: Adia; specificEpithet: cinerella; scientificNameAuthorship: (Fallén, 1825); **Location:** country: Pakistan; countryCode: PK; stateProvince: Balochistan; county: Ziatar District; municipality: Ziarat; decimalLatitude: 30.400139; decimalLongitude: 67.726806; geodeticDatum: WGS84; **Identification:** identifiedBy: Qaiser Khan; **Event:** eventDate: 2019-04-22
- **Type status:** Other material. **Occurrence:** recordedBy: Qaiser Khan; individualCount: 2; occurrenceID: 80FDDEC1-DECA-57C9-B6B1-D975371B16D9; **Taxon:** scientificName: Adiacinerella (Fallén, 1825); order: Diptera; family: Anthomyiidae; genus: Adia; specificEpithet: cinerella; scientificNameAuthorship: (Fallén, 1825); **Location:** country: Pakistan; countryCode: PK; stateProvince: Balochistan; county: Ziatar District; municipality: Ziarat; decimalLatitude: 30.400139; decimalLongitude: 67.726806; geodeticDatum: WGS84; **Identification:** identifiedBy: Qaiser Khan; **Event:** eventDate: 2019-04-29
- **Type status:** Other material. **Occurrence:** recordedBy: Qaiser Khan; individualCount: 8; occurrenceID: BA0A42C0-A568-5AB6-A3DF-19BE6D04E324; **Taxon:** scientificName: Adiacinerella (Fallén, 1825); order: Diptera; family: Anthomyiidae; genus: Adia; specificEpithet: cinerella; scientificNameAuthorship: (Fallén, 1825); **Location:** country: Pakistan; countryCode: PK; stateProvince: Balochistan; county: Ziatar District; municipality: Ziarat; decimalLatitude: 30.400139; decimalLongitude: 67.726806; geodeticDatum: WGS84; **Identification:** identifiedBy: Qaiser Khan; **Event:** eventDate: 2019-05-27
- **Type status:** Other material. **Occurrence:** recordedBy: Qaiser Khan; individualCount: 2; occurrenceID: 35942A4F-F612-50D6-BD19-47BE73B119E2; **Taxon:** scientificName: Adiacinerella (Fallén, 1825); order: Diptera; family: Anthomyiidae; genus: Adia; specificEpithet: cinerella; scientificNameAuthorship: (Fallén, 1825); **Location:** country: Pakistan; countryCode: PK; stateProvince: Balochistan; county: Ziatar District; municipality: Ziarat; decimalLatitude: 30.400139; decimalLongitude: 67.726806; geodeticDatum: WGS84; **Identification:** identifiedBy: Qaiser Khan; **Event:** eventDate: 2019-06-10
- **Type status:** Other material. **Occurrence:** recordedBy: Qaiser Khan; individualCount: 3; occurrenceID: 63B8B9EA-AE74-5B59-976F-4912FD96980B; **Taxon:** scientificName: Adiacinerella (Fallén, 1825); order: Diptera; family: Anthomyiidae; genus: Adia; specificEpithet: cinerella; scientificNameAuthorship: (Fallén, 1825); **Location:** country: Pakistan; countryCode: PK; stateProvince: Balochistan; county: Ziatar District; municipality: Ziarat; decimalLatitude: 30.400139; decimalLongitude: 67.726806; geodeticDatum: WGS84; **Identification:** identifiedBy: Qaiser Khan; **Event:** eventDate: 2019-06-17
- **Type status:** Other material. **Occurrence:** recordedBy: Qaiser Khan; individualCount: 9; occurrenceID: 9C03F11F-C0EE-5828-9801-6EB9E3BAF1F1; **Taxon:** scientificName: Adiacinerella (Fallén, 1825); order: Diptera; family: Anthomyiidae; genus: Adia; specificEpithet: cinerella; scientificNameAuthorship: (Fallén, 1825); **Location:** country: Pakistan; countryCode: PK; stateProvince: Balochistan; county: Ziatar District; municipality: Ziarat; decimalLatitude: 30.400139; decimalLongitude: 67.726806; geodeticDatum: WGS84; **Identification:** identifiedBy: Qaiser Khan; **Event:** eventDate: 2019-06-24
- **Type status:** Other material. **Occurrence:** recordedBy: Qaiser Khan; individualCount: 1; occurrenceID: C60780D7-CCE4-54F8-A77D-E704AC217602; **Taxon:** scientificName: Adiacinerella (Fallén, 1825); order: Diptera; family: Anthomyiidae; genus: Adia; specificEpithet: cinerella; scientificNameAuthorship: (Fallén, 1825); **Location:** country: Pakistan; countryCode: PK; stateProvince: Balochistan; county: Ziatar District; municipality: Ziarat; decimalLatitude: 30.400139; decimalLongitude: 67.726806; geodeticDatum: WGS84; **Identification:** identifiedBy: Qaiser Khan; **Event:** eventDate: 2019-07-15
- **Type status:** Other material. **Occurrence:** recordedBy: Qaiser Khan; individualCount: 29; occurrenceID: B612C709-C7BB-547D-B770-0FA52015A194; **Taxon:** scientificName: Adiacinerella (Fallén, 1825); order: Diptera; family: Anthomyiidae; genus: Adia; specificEpithet: cinerella; scientificNameAuthorship: (Fallén, 1825); **Location:** country: Pakistan; countryCode: PK; stateProvince: v; county: Ziatar District; municipality: Ziarat; decimalLatitude: 30.400139; decimalLongitude: 67.726806; geodeticDatum: WGS84; **Identification:** identifiedBy: Qaiser Khan; **Event:** eventDate: 2019-07-22
- **Type status:** Other material. **Occurrence:** recordedBy: Qaiser Khan; individualCount: 3; occurrenceID: 54D56423-FB7A-5354-A222-076012965CD9; **Taxon:** scientificName: Adiacinerella (Fallén, 1825); order: Diptera; family: Anthomyiidae; genus: Adia; specificEpithet: cinerella; scientificNameAuthorship: (Fallén, 1825); **Location:** country: Pakistan; countryCode: PK; stateProvince: Balochistan; county: Ziatar District; municipality: Ziarat; decimalLatitude: 30.400139; decimalLongitude: 67.726806; geodeticDatum: WGS84; **Identification:** identifiedBy: Qaiser Khan; **Event:** eventDate: 2019-07-29
- **Type status:** Other material. **Occurrence:** recordedBy: Qaiser Khan; individualCount: 23; occurrenceID: E3C462C6-BA7A-5F8D-B64E-B994693488A5; **Taxon:** scientificName: Adiacinerella (Fallén, 1825); order: Diptera; family: Anthomyiidae; genus: Adia; specificEpithet: cinerella; scientificNameAuthorship: (Fallén, 1825); **Location:** country: Pakistan; countryCode: PK; stateProvince: Balochistan; county: Ziatar District; municipality: Ziarat; decimalLatitude: 30.400139; decimalLongitude: 67.726806; geodeticDatum: WGS84; **Identification:** identifiedBy: Qaiser Khan; **Event:** eventDate: 2019-08-26
- **Type status:** Other material. **Occurrence:** recordedBy: Qaiser Khan; individualCount: 3; occurrenceID: 7C52E644-68BC-594A-8CBD-679BAD4D1D5F; **Taxon:** scientificName: Adiacinerella (Fallén, 1825); order: Diptera; family: Anthomyiidae; genus: Adia; specificEpithet: cinerella; scientificNameAuthorship: (Fallén, 1825); **Location:** country: Pakistan; countryCode: PK; stateProvince: Balochistan; county: Ziatar District; municipality: Ziarat; decimalLatitude: 30.400139; decimalLongitude: 67.726806; geodeticDatum: WGS84; **Identification:** identifiedBy: Qaiser Khan; **Event:** eventDate: 2019-09-23
- **Type status:** Other material. **Occurrence:** recordedBy: Qaiser Khan; individualCount: 7; occurrenceID: 4CFDBB40-9C1F-5E25-8F2F-94F334FE28FB; **Taxon:** scientificName: Adiacinerella (Fallén, 1825); order: Diptera; family: Anthomyiidae; genus: Adia; specificEpithet: cinerella; scientificNameAuthorship: (Fallén, 1825); **Location:** country: Pakistan; countryCode: PK; stateProvince: Balochistan; county: Ziatar District; municipality: Ziarat; decimalLatitude: 30.400139; decimalLongitude: 67.726806; geodeticDatum: WGS84; **Identification:** identifiedBy: Qaiser Khan; **Event:** eventDate: 2019-09-30
- **Type status:** Other material. **Occurrence:** recordedBy: Qaiser Khan; individualCount: 1; occurrenceID: 9C42FDD0-BBC3-5885-97E5-7B043C79F736; **Taxon:** scientificName: Adiacinerella (Fallén, 1825); order: Diptera; family: Anthomyiidae; genus: Adia; specificEpithet: cinerella; scientificNameAuthorship: (Fallén, 1825); **Location:** country: Pakistan; countryCode: PK; stateProvince: Balochistan; county: Ziatar District; municipality: Ziarat; decimalLatitude: 30.400139; decimalLongitude: 67.726806; geodeticDatum: WGS84; **Identification:** identifiedBy: Qaiser Khan; **Event:** eventDate: 2019-10-07
- **Type status:** Other material. **Occurrence:** recordedBy: Qaiser Khan; individualCount: 5; occurrenceID: 38A032E0-E0D2-52F8-A6D4-B27953F0A4EE; **Taxon:** scientificName: Adiacinerella (Fallén, 1825); order: Diptera; family: Anthomyiidae; genus: Adia; specificEpithet: cinerella; scientificNameAuthorship: (Fallén, 1825); **Location:** country: Pakistan; countryCode: PK; stateProvince: Balochistan; county: Ziatar District; municipality: Ziarat; decimalLatitude: 30.400139; decimalLongitude: 67.726806; geodeticDatum: WGS84; **Identification:** identifiedBy: Qaiser Khan; **Event:** eventDate: 2019-10-14
- **Type status:** Other material. **Occurrence:** recordedBy: Qaiser Khan; individualCount: 1; occurrenceID: 4C189818-2FB1-505B-8D35-C26B64F1C9E1; **Taxon:** scientificName: Adiacinerella (Fallén, 1825); order: Diptera; family: Anthomyiidae; genus: Adia; specificEpithet: cinerella; scientificNameAuthorship: (Fallén, 1825); **Location:** country: Pakistan; countryCode: PK; stateProvince: Balochistan; county: Ziatar District; municipality: Ziarat; decimalLatitude: 30.400139; decimalLongitude: 67.726806; geodeticDatum: WGS84; **Identification:** identifiedBy: Qaiser Khan; **Event:** eventDate: 2019-11-11
- **Type status:** Other material. **Occurrence:** recordedBy: Qaiser Khan; individualCount: 2; occurrenceID: 89955B63-9058-5B45-8DAD-7ED7B3ADB1B1; **Taxon:** scientificName: Adiacinerella (Fallén, 1825); order: Diptera; family: Anthomyiidae; genus: Adia; specificEpithet: cinerella; scientificNameAuthorship: (Fallén, 1825); **Location:** country: Pakistan; countryCode: PK; stateProvince: Balochistan; county: Ziatar District; municipality: Ziarat; decimalLatitude: 30.400139; decimalLongitude: 67.726806; geodeticDatum: WGS84; **Identification:** identifiedBy: Qaiser Khan; **Event:** eventDate: 2019-11-18

#### Distribution

Commonly found across the Holarctic and Oriental territories (Pont 2012, Szalanski et al. 2004, Suwa 2011, Tao et al. 2000).

#### Notes

**Figure 2.**
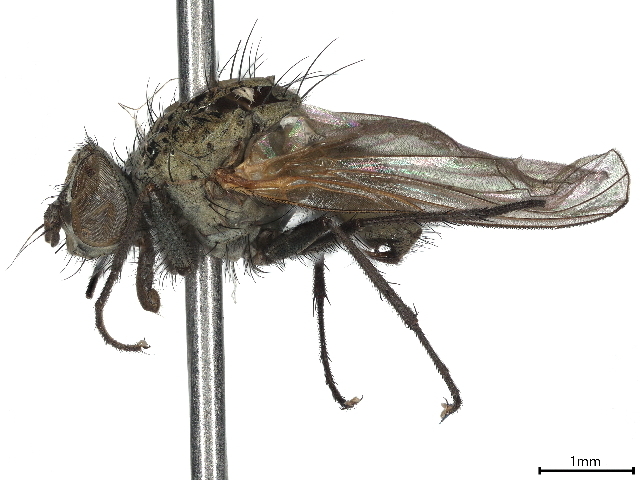
*Adiacinerellas* (Fallén, 1825).

This species is the first country record having agricultural importance (Kara and Ullusoy 2016). This species has been documented for the first time in the study area. It is actively found from March to November (Fig. 2).

### Atherigona soccata

Rondani, 1871

3BA91167-34DC-5B93-BD9E-D8B080EFD92D

OR685684

#### Materials

- **Type status:** Other material. **Occurrence:** recordedBy: Qaiser Khan; individualCount: 1; occurrenceID: 34161F35-E30B-5222-8549-447C131A7109; **Taxon:** scientificName: Atherigonasoccata Rondani 1871; order: Diptera; family: Muscidae; genus: Atherigona; specificEpithet: soccata; scientificNameAuthorship: Rondani, 1871; **Location:** country: Pakistan; countryCode: PK; stateProvince: Balochistan; county: Ziatar District; municipality: Ziarat; decimalLatitude: 30.400139; decimalLongitude: 67.726806; geodeticDatum: WGS84; **Identification:** identifiedBy: Qaiser Khan; **Event:** eventDate: 2019-04-22
- **Type status:** Other material. **Occurrence:** recordedBy: Qaiser Khan; individualCount: 1; occurrenceID: 6FA6ECD4-CABE-55B2-9E08-95BDADC7531E; **Taxon:** scientificName: Atherigonasoccata Rondani 1871; order: Diptera; family: Muscidae; genus: Atherigona; specificEpithet: soccata; scientificNameAuthorship: Rondani, 1871; **Location:** country: Pakistan; countryCode: PK; stateProvince: Balochistan; county: Ziatar District; municipality: Ziarat; decimalLatitude: 30.400139; decimalLongitude: 67.726806; geodeticDatum: WGS84; **Identification:** identifiedBy: Qaiser Khan; **Event:** eventDate: 2019-05-13
- **Type status:** Other material. **Occurrence:** recordedBy: Qaiser Khan; individualCount: 5; occurrenceID: 321AF9BE-46CB-5A50-BC24-D883FCB6B94B; **Taxon:** scientificName: Atherigonasoccata Rondani 1871; order: Diptera; family: Muscidae; genus: Atherigona; specificEpithet: soccata; scientificNameAuthorship: Rondani, 1871; **Location:** country: Pakistan; countryCode: PK; stateProvince: Balochistan; county: Ziatar District; municipality: Ziarat; decimalLatitude: 30.400139; decimalLongitude: 67.726806; geodeticDatum: WGS84; **Identification:** identifiedBy: Qaiser Khan; **Event:** eventDate: 2019-06-26
- **Type status:** Other material. **Occurrence:** recordedBy: Qaiser Khan; individualCount: 1; occurrenceID: 81B16E32-9204-524A-B3D5-E2C46D453D19; **Taxon:** scientificName: Atherigonasoccata Rondani 1871; order: Diptera; family: Muscidae; genus: Atherigona; specificEpithet: soccata; scientificNameAuthorship: Rondani, 1871; **Location:** country: Pakistan; countryCode: PK; stateProvince: Balochistan; county: Ziatar District; municipality: Ziarat; decimalLatitude: 30.400139; decimalLongitude: 67.726806; geodeticDatum: WGS84; **Identification:** identifiedBy: Qaiser Khan; **Event:** eventDate: 2019-07-22
- **Type status:** Other material. **Occurrence:** recordedBy: Qaiser Khan; individualCount: 9; occurrenceID: D0EE79D0-68DF-535C-90FC-177051EB53E2; **Taxon:** scientificName: Atherigonasoccata Rondani 1871; order: Diptera; family: Muscidae; genus: Atherigona; specificEpithet: soccata; scientificNameAuthorship: Rondani, 1871; **Location:** country: Pakistan; countryCode: PK; stateProvince: Balochistan; county: Ziatar District; municipality: Ziarat; decimalLatitude: 30.400139; decimalLongitude: 67.726806; geodeticDatum: WGS84; **Identification:** identifiedBy: Qaiser Khan; **Event:** eventDate: 2019-07-29
- **Type status:** Other material. **Occurrence:** recordedBy: Qaiser Khan; individualCount: 7; occurrenceID: 059FEEFA-ACD4-52BB-A05C-9EBACBFFBFA9; **Taxon:** scientificName: Atherigonasoccata Rondani 1871; order: Diptera; family: Muscidae; genus: Atherigona; specificEpithet: soccata; scientificNameAuthorship: Rondani, 1871; **Location:** country: Pakistan; countryCode: PK; stateProvince: Balochistan; county: Ziatar District; municipality: Ziarat; decimalLatitude: 30.400139; decimalLongitude: 67.726806; geodeticDatum: WGS84; **Identification:** identifiedBy: Qaiser Khan; **Event:** eventDate: 2019-08-19
- **Type status:** Other material. **Occurrence:** recordedBy: Qaiser Khan; individualCount: 5; occurrenceID: 43A39D67-3FBE-5690-A0D9-3587238B455F; **Taxon:** scientificName: Atherigonasoccata Rondani 1871; order: Diptera; family: Muscidae; genus: Atherigona; specificEpithet: soccata; scientificNameAuthorship: Rondani, 1871; **Location:** country: Pakistan; countryCode: PK; stateProvince: Balochistan; county: Ziatar District; municipality: Ziarat; decimalLatitude: 30.400139; decimalLongitude: 67.726806; geodeticDatum: WGS84; **Identification:** identifiedBy: Qaiser Khan; **Event:** eventDate: 2019-09-02
- **Type status:** Other material. **Occurrence:** recordedBy: Qaiser Khan; individualCount: 1; occurrenceID: 1E8FD172-2269-5BCA-A4C5-164D9A03D35B; **Taxon:** scientificName: Atherigonasoccata Rondani 1871; order: Diptera; family: Muscidae; genus: Atherigona; specificEpithet: soccata; scientificNameAuthorship: Rondani, 1871; **Location:** country: Pakistan; countryCode: PK; stateProvince: Balochistan; county: Ziatar District; municipality: Ziarat; decimalLatitude: 30.400139; decimalLongitude: 67.726806; geodeticDatum: WGS84; **Identification:** identifiedBy: Qaiser Khan; **Event:** eventDate: 2019-09-30
- **Type status:** Other material. **Occurrence:** recordedBy: Qaiser Khan; individualCount: 4; occurrenceID: B7BE7022-71AC-5E00-B1BB-A1CCE1E1BFFE; **Taxon:** scientificName: Atherigonasoccata Rondani 1871; order: Diptera; family: Muscidae; genus: Atherigona; specificEpithet: soccata; scientificNameAuthorship: Rondani, 1871; **Location:** country: Pakistan; countryCode: PK; stateProvince: Balochistan; county: Ziatar District; municipality: Ziarat; decimalLatitude: 30.400139; decimalLongitude: 67.726806; geodeticDatum: WGS84; **Identification:** identifiedBy: Qaiser Khan; **Event:** eventDate: 2019-10-21
- **Type status:** Other material. **Occurrence:** recordedBy: Qaiser Khan; individualCount: 3; occurrenceID: 5CAE0C90-C4C9-55D0-94D9-C31A40259521; **Taxon:** scientificName: Atherigonasoccata Rondani 1871; order: Diptera; family: Muscidae; genus: Atherigona; specificEpithet: soccata; scientificNameAuthorship: Rondani, 1871; **Location:** country: Pakistan; countryCode: PK; stateProvince: Balochistan; county: Ziatar District; municipality: Ziarat; decimalLatitude: 30.400139; decimalLongitude: 67.726806; geodeticDatum: WGS84; **Identification:** identifiedBy: Qaiser Khan; **Event:** eventDate: 2019-11-18

#### Distribution

Species widely distributed, recorded the Afrotropical egion, China (Guangdong), India, Middle East, Myanmar, Nepal, North Africa, Philippines, southern Europe and Thailand (Pont and Magpayo 1995, Kalaisekar et al. 2016); in Pakistan, it has been recorded by (Arif et al. (2013), Khaliq et al. (2022), Khan et al. (2022))

#### Notes

**Figure 3.**
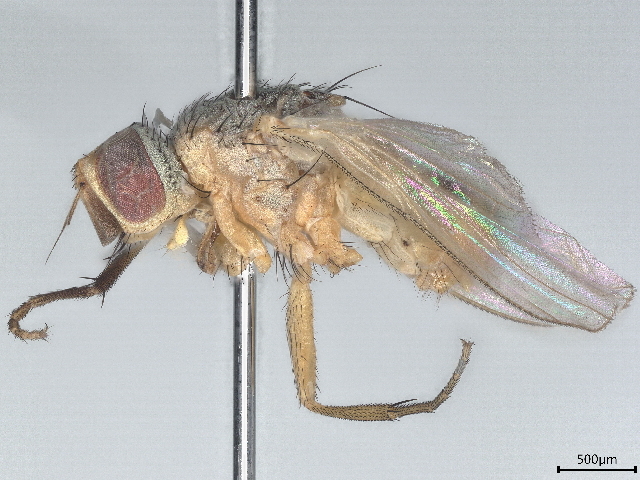
*Atherigonasoccata* (Schiner, 1868).

The sorgum shoot fly is a major pest to grain sorghum, particularly in regions where it is the major rainfed crop (Khaliq et al. 2022). This insect has a high rate of occurance after rainfall, making it a highly destructive species in locations all over the world (Edde 2021). Khaliq et al. (2022) reported the presence of this species in Punjab, Pakistan. This species was observed from April to October (Fig. 3).

### Atherigona varia

(Meigen, 1826)

34816C89-B441-5586-909F-44532A0C7358

OR685685

OR685686

#### Materials

- **Type status:** Other material. **Occurrence:** recordedBy: Qaiser Khan; individualCount: 1; occurrenceID: 99180FD9-768B-5133-99EF-7D979AB5BF94; **Taxon:** scientificName: Atherigonavaria (Meigen, 1826); order: Diptera; family: Muscidae; genus: Atherigona; specificEpithet: varia; scientificNameAuthorship: (Meigen, 1826); **Location:** country: Pakistan; countryCode: PK; stateProvince: Balochistan; county: Ziatar District; municipality: Ziarat; decimalLatitude: 30.400139; decimalLongitude: 67.726806; geodeticDatum: WGS84; **Identification:** identifiedBy: Qaiser Khan; **Event:** eventDate: 2019-03-04
- **Type status:** Other material. **Occurrence:** recordedBy: Qaiser Khan; individualCount: 3; occurrenceID: 6C15BB74-F589-561E-BEB7-EF3027E6AD70; **Taxon:** scientificName: Atherigonavaria (Meigen, 1826); order: Diptera; family: Muscidae; genus: Atherigona; specificEpithet: varia; scientificNameAuthorship: (Meigen, 1826); **Location:** country: Pakistan; countryCode: PK; stateProvince: Balochistan; county: Ziatar District; municipality: Ziarat; decimalLatitude: 30.400139; decimalLongitude: 67.726806; geodeticDatum: WGS84; **Identification:** identifiedBy: Qaiser Khan; **Event:** eventDate: 2019-04-22
- **Type status:** Other material. **Occurrence:** recordedBy: Qaiser Khan; individualCount: 1; occurrenceID: 118316F8-FA81-5937-949E-76FA311E4A15; **Taxon:** scientificName: Atherigonavaria (Meigen, 1826); order: Diptera; family: Muscidae; genus: Atherigona; specificEpithet: varia; scientificNameAuthorship: (Meigen, 1826); **Location:** country: Pakistan; countryCode: PK; stateProvince: Balochistan; county: Ziatar District; municipality: Ziarat; decimalLatitude: 30.400139; decimalLongitude: 67.726806; geodeticDatum: WGS84; **Identification:** identifiedBy: Qaiser Khan; **Event:** eventDate: 2019-04-29
- **Type status:** Other material. **Occurrence:** recordedBy: Qaiser Khan; individualCount: 7; occurrenceID: 8CFEFFB1-BB4B-5E4A-B946-31E04C1E654B; **Taxon:** scientificName: Atherigonavaria (Meigen, 1826); order: Diptera; family: Muscidae; genus: Atherigona; specificEpithet: varia; scientificNameAuthorship: (Meigen, 1826); **Location:** country: Pakistan; countryCode: PK; stateProvince: Balochistan; county: Ziatar District; municipality: Ziarat; decimalLatitude: 30.400139; decimalLongitude: 67.726806; geodeticDatum: WGS84; **Identification:** identifiedBy: Qaiser Khan; **Event:** eventDate: 2019-05-22
- **Type status:** Other material. **Occurrence:** recordedBy: Qaiser Khan; individualCount: 1; occurrenceID: EDD87C70-673B-5A50-B03E-3F66DDFB45AF; **Taxon:** scientificName: Atherigonavaria (Meigen, 1826); order: Diptera; family: Muscidae; genus: Atherigona; specificEpithet: varia; scientificNameAuthorship: (Meigen, 1826); **Location:** country: Pakistan; countryCode: PK; stateProvince: Balochistan; county: Ziatar District; municipality: Ziarat; decimalLatitude: 30.400139; decimalLongitude: 67.726806; geodeticDatum: WGS84; **Identification:** identifiedBy: Qaiser Khan; **Event:** eventDate: 2019-05-27
- **Type status:** Other material. **Occurrence:** recordedBy: Qaiser Khan; individualCount: 1; occurrenceID: 15AF3B5D-49A4-51F4-B55D-367F00AD37CA; **Taxon:** scientificName: Atherigonavaria (Meigen, 1826); order: Diptera; family: Muscidae; genus: Atherigona; specificEpithet: varia; scientificNameAuthorship: (Meigen, 1826); **Location:** country: Pakistan; countryCode: PK; stateProvince: Balochistan; county: Ziatar District; municipality: Ziarat; decimalLatitude: 30.400139; decimalLongitude: 67.726806; geodeticDatum: WGS84; **Identification:** identifiedBy: Qaiser Khan; **Event:** eventDate: 2019-06-24
- **Type status:** Other material. **Occurrence:** recordedBy: Qaiser Khan; individualCount: 3; occurrenceID: 726D269F-81F8-57F4-B54A-8A07D7464B8A; **Taxon:** scientificName: Atherigonavaria (Meigen, 1826); order: Diptera; family: Muscidae; genus: Atherigona; specificEpithet: varia; scientificNameAuthorship: (Meigen, 1826); **Location:** country: Pakistan; countryCode: PK; stateProvince: Balochistan; county: Ziatar District; municipality: Ziarat; decimalLatitude: 30.400139; decimalLongitude: 67.726806; geodeticDatum: WGS84; **Identification:** identifiedBy: Qaiser Khan; **Event:** eventDate: 2019-07-03
- **Type status:** Other material. **Occurrence:** recordedBy: Qaiser Khan; individualCount: 14; occurrenceID: A9A50D78-01C7-5EDF-8376-B40603DE6525; **Taxon:** scientificName: Atherigonavaria (Meigen, 1826); order: Diptera; family: Muscidae; genus: Atherigona; specificEpithet: varia; scientificNameAuthorship: (Meigen, 1826); **Location:** country: Pakistan; countryCode: PK; stateProvince: Balochistan; county: Ziatar District; municipality: Ziarat; decimalLatitude: 30.400139; decimalLongitude: 67.726806; geodeticDatum: WGS84; **Identification:** identifiedBy: Qaiser Khan; **Event:** eventDate: 2019-07-22
- **Type status:** Other material. **Occurrence:** recordedBy: Qaiser Khan; individualCount: 3; occurrenceID: 12107E24-1930-58D0-BD95-9DAD100E92B5; **Taxon:** scientificName: Atherigonavaria (Meigen, 1826); order: Diptera; family: Muscidae; genus: Atherigona; specificEpithet: varia; scientificNameAuthorship: (Meigen, 1826); **Location:** country: Pakistan; countryCode: PK; stateProvince: Balochistan; county: Ziatar District; municipality: Ziarat; decimalLatitude: 30.400139; decimalLongitude: 67.726806; geodeticDatum: WGS84; **Identification:** identifiedBy: Qaiser Khan; **Event:** eventDate: 2019-07-29
- **Type status:** Other material. **Occurrence:** recordedBy: Qaiser Khan; individualCount: 19; occurrenceID: D5DE031F-DF8E-5512-810A-364B66BEDADE; **Taxon:** scientificName: Atherigonavaria (Meigen, 1826); order: Diptera; family: Muscidae; genus: Atherigona; specificEpithet: varia; scientificNameAuthorship: (Meigen, 1826); **Location:** country: Pakistan; countryCode: PK; stateProvince: Balochistan; county: Ziatar District; municipality: Ziarat; decimalLatitude: 30.400139; decimalLongitude: 67.726806; geodeticDatum: WGS84; **Identification:** identifiedBy: Qaiser Khan; **Event:** eventDate: 2019-08-05
- **Type status:** Other material. **Occurrence:** recordedBy: Qaiser Khan; individualCount: 1; occurrenceID: 8A39CB25-B892-59B1-803C-9E479B4F4700; **Taxon:** scientificName: Atherigonavaria (Meigen, 1826); order: Diptera; family: Muscidae; genus: Atherigona; specificEpithet: varia; scientificNameAuthorship: (Meigen, 1826); **Location:** country: Pakistan; countryCode: PK; stateProvince: Balochistan; county: Ziatar District; municipality: Ziarat; decimalLatitude: 30.400139; decimalLongitude: 67.726806; geodeticDatum: WGS84; **Identification:** identifiedBy: Qaiser Khan; **Event:** eventDate: 2019-09-09
- **Type status:** Other material. **Occurrence:** recordedBy: Qaiser Khan; individualCount: 11; occurrenceID: 09731CDB-1EB8-5582-B4AA-C8340DC70EC0; **Taxon:** scientificName: Atherigonavaria (Meigen, 1826); order: Diptera; family: Muscidae; genus: Atherigona; specificEpithet: varia; scientificNameAuthorship: (Meigen, 1826); **Location:** country: Pakistan; countryCode: PK; stateProvince: Balochistan; county: Ziatar District; municipality: Ziarat; decimalLatitude: 30.400139; decimalLongitude: 67.726806; geodeticDatum: WGS84; **Identification:** identifiedBy: Qaiser Khan; **Event:** eventDate: 2019-09-30
- **Type status:** Other material. **Occurrence:** recordedBy: Qaiser Khan; individualCount: 7; occurrenceID: 80E26933-B20B-52C9-8131-21A19E9C28B9; **Taxon:** scientificName: Atherigonavaria (Meigen, 1826); order: Diptera; family: Muscidae; genus: Atherigona; specificEpithet: varia; scientificNameAuthorship: (Meigen, 1826); **Location:** country: Pakistan; countryCode: PK; stateProvince: Balochistan; county: Ziatar District; municipality: Ziarat; decimalLatitude: 30.400139; decimalLongitude: 67.726806; geodeticDatum: WGS84; **Identification:** identifiedBy: Qaiser Khan; **Event:** eventDate: 2019-10-17
- **Type status:** Other material. **Occurrence:** recordedBy: Qaiser Khan; individualCount: 5; occurrenceID: BB73AF91-CC4E-5653-94FB-32A7D85E5897; **Taxon:** scientificName: Atherigonavaria (Meigen, 1826); order: Diptera; family: Muscidae; genus: Atherigona; specificEpithet: varia; scientificNameAuthorship: (Meigen, 1826); **Location:** country: Pakistan; countryCode: PK; stateProvince: Balochistan; county: Ziatar District; municipality: Ziarat; decimalLatitude: 30.400139; decimalLongitude: 67.726806; geodeticDatum: WGS84; **Identification:** identifiedBy: Qaiser Khan; **Event:** eventDate: 2019-11-18

#### Distribution

**Figure 4.**
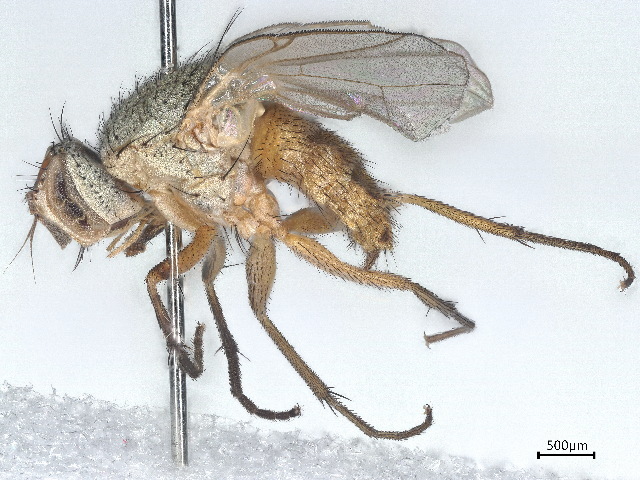
*Atherigonavaria* (Schiner, 1868).

The Middle East, outhern Europe and China (Pont 2018, Akmeşe et al. 2016). It has also been reported from Pakistan (Moiz and Naqvi 1968). Remarkably, this species has been newly documented in Ziarat (Fig. 4).

#### Notes

This species is a major pest of grass and grain crops (Deeming 2022). Its activity spans from March to November within the study area, fading away from late November until mid-March.

### Chironomus dorsalis

Meigen, 1818

E914612F-0125-5227-81CA-3E3648B8F26D

OR685687

#### Materials

- **Type status:** Other material. **Occurrence:** recordedBy: Qaiser Khan; individualCount: 3; occurrenceID: 3823C149-2814-56B4-80FD-39A5B19C1A04; **Taxon:** scientificName: Chironomusdorsalis Meigen, 1818; order: Diptera; family: Chironomidae; genus: Chironomus; specificEpithet: dorsalis; scientificNameAuthorship: Meigen, 1818; **Location:** country: Pakistan; countryCode: PK; stateProvince: Balochistan; county: Ziatar District; municipality: Ziarat; decimalLatitude: 30.400139; decimalLongitude: 67.726806; geodeticDatum: WGS84; **Identification:** identifiedBy: Qaiser Khan; **Event:** eventDate: 2019-07-03
- **Type status:** Other material. **Occurrence:** recordedBy: Qaiser Khan; individualCount: 9; occurrenceID: 0CD6755A-C5CC-58D2-BDE6-F628C0B36C79; **Taxon:** scientificName: Chironomusdorsalis Meigen, 1818; order: Diptera; family: Chironomidae; genus: Chironomus; specificEpithet: dorsalis; scientificNameAuthorship: Meigen, 1818; **Location:** country: Pakistan; countryCode: PK; stateProvince: Balochistan; county: Ziatar District; municipality: Ziarat; decimalLatitude: 30.400139; decimalLongitude: 67.726806; geodeticDatum: WGS84; **Identification:** identifiedBy: Qaiser Khan; **Event:** eventDate: 2019-08-12
- **Type status:** Other material. **Occurrence:** recordedBy: Qaiser Khan; individualCount: 1; occurrenceID: E973AD77-2D04-5EE7-A04C-80384D86868D; **Taxon:** scientificName: Chironomusdorsalis Meigen, 1818; order: Diptera; family: Chironomidae; genus: Chironomus; specificEpithet: dorsalis; scientificNameAuthorship: Meigen, 1818; **Location:** country: Pakistan; countryCode: PK; stateProvince: Balochistan; county: Ziatar District; municipality: Ziarat; decimalLatitude: 30.400139; decimalLongitude: 67.726806; geodeticDatum: WGS84; **Identification:** identifiedBy: Qaiser Khan; **Event:** eventDate: 2019-09-09

#### Distribution

**Figure 5.**
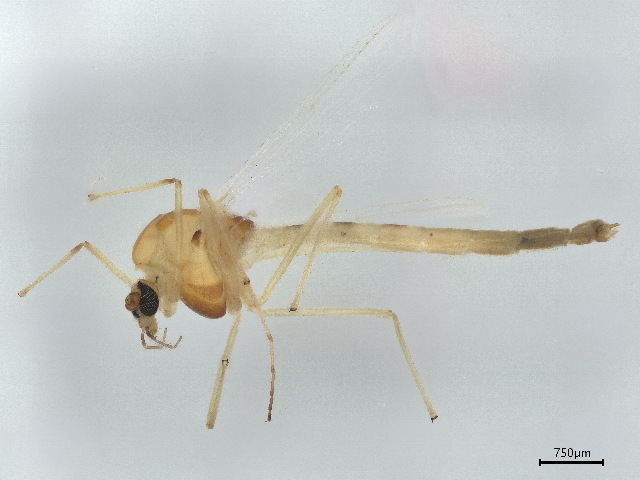
*Chironomusdorsalis* (Meigen, 1818)

This species has been documented in both Asia and North America, reflecting its almost cosmopolitan distribution (Yan et al. 2021, System 2023, Na et al. 2010, Kiknadze et al. 2008). This species is documented for the first time in Ziarat (Fig. 5).

#### Notes

This species is considered a good bioindicator (Kiknadze et al. 2008). The survival duration of this species is short-lived, appearing from July to the end of September.

### Eupeodes corollae

(Fabricius, 1794)

FB68CA0C-78A2-522D-A613-08BA14CBA533

OR685690

#### Materials

- **Type status:** Other material. **Occurrence:** recordedBy: Qaiser Khan; individualCount: 2; occurrenceID: B138F362-9272-5450-B526-355FC7EDD93F; **Taxon:** scientificName: Eupeodescorollae (Fabricius, 1794); order: Diptera; family: Syrphidae; genus: Eupeodes; specificEpithet: corollae; scientificNameAuthorship: (Fabricius, 1794); **Location:** country: Pakistan; countryCode: PK; stateProvince: Balochistan; county: Ziatar District; municipality: Ziarat; decimalLatitude: 30.400139; decimalLongitude: 67.726806; geodeticDatum: WGS84; **Identification:** identifiedBy: Qaiser Khan; **Event:** eventDate: 2019-03-04
- **Type status:** Other material. **Occurrence:** recordedBy: Qaiser Khan; individualCount: 1; occurrenceID: FC91833E-1C00-5827-BA6C-CA053EACBBB2; **Taxon:** scientificName: Eupeodescorollae (Fabricius, 1794); order: Diptera; family: Syrphidae; genus: Eupeodes; specificEpithet: corollae; scientificNameAuthorship: (Fabricius, 1794); **Location:** country: Pakistan; countryCode: PK; stateProvince: Balochistan; county: Ziatar District; municipality: Ziarat; decimalLatitude: 30.400139; decimalLongitude: 67.726806; geodeticDatum: WGS84; **Identification:** identifiedBy: Qaiser Khan; **Event:** eventDate: 2019-03-11
- **Type status:** Other material. **Occurrence:** recordedBy: Qaiser Khan; individualCount: 5; occurrenceID: F0231F05-EA39-524A-B538-D86938FE3A27; **Taxon:** scientificName: Eupeodescorollae (Fabricius, 1794); order: Diptera; family: Syrphidae; genus: Eupeodes; specificEpithet: corollae; scientificNameAuthorship: (Fabricius, 1794); **Location:** country: Pakistan; countryCode: PK; stateProvince: Balochistan; county: Ziatar District; municipality: Ziarat; decimalLatitude: 30.400139; decimalLongitude: 67.726806; geodeticDatum: WGS84; **Identification:** identifiedBy: Qaiser Khan; **Event:** eventDate: 2019-03-25
- **Type status:** Other material. **Occurrence:** recordedBy: Qaiser Khan; individualCount: 25; occurrenceID: 0BC5FBF6-A942-5A1A-9F27-3F0A2616CBF5; **Taxon:** scientificName: Eupeodescorollae (Fabricius, 1794); order: Diptera; family: Syrphidae; genus: Eupeodes; specificEpithet: corollae; scientificNameAuthorship: (Fabricius, 1794); **Location:** country: Pakistan; countryCode: PK; stateProvince: Balochistan; county: Ziatar District; municipality: Ziarat; decimalLatitude: 30.400139; decimalLongitude: 67.726806; geodeticDatum: WGS84; **Identification:** identifiedBy: Qaiser Khan; **Event:** eventDate: 2019-04-08
- **Type status:** Other material. **Occurrence:** recordedBy: Qaiser Khan; individualCount: 9; occurrenceID: 61C102FC-4BF2-5A18-90AA-774ACBF6B260; **Taxon:** scientificName: Eupeodescorollae (Fabricius, 1794); order: Diptera; family: Syrphidae; genus: Eupeodes; specificEpithet: corollae; scientificNameAuthorship: (Fabricius, 1794); **Location:** country: Pakistan; countryCode: PK; stateProvince: Balochistan; county: Ziatar District; municipality: Ziarat; decimalLatitude: 30.400139; decimalLongitude: 67.726806; geodeticDatum: WGS84; **Identification:** identifiedBy: Qaiser Khan; **Event:** eventDate: 2019-04-15
- **Type status:** Other material. **Occurrence:** recordedBy: Qaiser Khan; individualCount: 123; occurrenceID: 565449F0-70E5-5CEB-8960-74ED5B4E6B54; **Taxon:** scientificName: Eupeodescorollae (Fabricius, 1794); order: Diptera; family: Syrphidae; genus: Eupeodes; specificEpithet: corollae; scientificNameAuthorship: (Fabricius, 1794); **Location:** country: Pakistan; countryCode: PK; stateProvince: Balochistan; county: Ziatar District; municipality: Ziarat; decimalLatitude: 30.400139; decimalLongitude: 67.726806; geodeticDatum: WGS84; **Identification:** identifiedBy: Qaiser Khan; **Event:** eventDate: 2019-04-22
- **Type status:** Other material. **Occurrence:** recordedBy: Qaiser Khan; individualCount: 8; occurrenceID: C621B73E-78CF-5A99-B0B2-5C1820B3A9B0; **Taxon:** scientificName: Eupeodescorollae (Fabricius, 1794); order: Diptera; family: Syrphidae; genus: Eupeodes; specificEpithet: corollae; scientificNameAuthorship: (Fabricius, 1794); **Location:** country: Pakistan; countryCode: PK; stateProvince: Balochistan; county: Ziatar District; municipality: Ziarat; decimalLatitude: 30.400139; decimalLongitude: 67.726806; geodeticDatum: WGS84; **Identification:** identifiedBy: Qaiser Khan; **Event:** eventDate: 2019-04-29
- **Type status:** Other material. **Occurrence:** recordedBy: Qaiser Khan; individualCount: 135; occurrenceID: D589EB68-39BC-5F57-94E5-349E1A642F24; **Taxon:** scientificName: Eupeodescorollae (Fabricius, 1794); order: Diptera; family: Syrphidae; genus: Eupeodes; specificEpithet: corollae; scientificNameAuthorship: (Fabricius, 1794); **Location:** country: Pakistan; countryCode: PK; stateProvince: Balochistan; county: Ziatar District; municipality: Ziarat; decimalLatitude: 30.400139; decimalLongitude: 67.726806; geodeticDatum: WGS84; **Identification:** identifiedBy: Qaiser Khan; **Event:** eventDate: 2019-05-20
- **Type status:** Other material. **Occurrence:** recordedBy: Qaiser Khan; individualCount: 52; occurrenceID: 9CD3CA2A-3DBB-56F1-B3FB-33592F8AEDEE; **Taxon:** scientificName: Eupeodescorollae (Fabricius, 1794); order: Diptera; family: Syrphidae; genus: Eupeodes; specificEpithet: corollae; scientificNameAuthorship: (Fabricius, 1794); **Location:** country: Pakistan; countryCode: PK; stateProvince: Balochistan; county: Ziatar District; municipality: Ziarat; decimalLatitude: 30.400139; decimalLongitude: 67.726806; geodeticDatum: WGS84; **Identification:** identifiedBy: Qaiser Khan; **Event:** eventDate: 2019-05-27
- **Type status:** Other material. **Occurrence:** recordedBy: Qaiser Khan; individualCount: 17; occurrenceID: EC81AF13-9D9E-5588-A990-3F533A437555; **Taxon:** scientificName: Eupeodescorollae (Fabricius, 1794); order: Diptera; family: Syrphidae; genus: Eupeodes; specificEpithet: corollae; scientificNameAuthorship: (Fabricius, 1794); **Location:** country: Pakistan; countryCode: PK; stateProvince: Balochistan; county: Ziatar District; municipality: Ziarat; decimalLatitude: 30.400139; decimalLongitude: 67.726806; geodeticDatum: WGS84; **Identification:** identifiedBy: Qaiser Khan; **Event:** eventDate: 2019-06-03
- **Type status:** Other material. **Occurrence:** recordedBy: Qaiser Khan; individualCount: 19; occurrenceID: 73B6FB3D-07BE-5354-B6D9-774629A4CAC2; **Taxon:** scientificName: Eupeodescorollae (Fabricius, 1794); order: Diptera; family: Syrphidae; genus: Eupeodes; specificEpithet: corollae; scientificNameAuthorship: (Fabricius, 1794); **Location:** country: Pakistan; countryCode: PK; stateProvince: Balochistan; county: Ziatar District; municipality: Ziarat; decimalLatitude: 30.400139; decimalLongitude: 67.726806; geodeticDatum: WGS84; **Identification:** identifiedBy: Qaiser Khan; **Event:** eventDate: 2019-06-24
- **Type status:** Other material. **Occurrence:** recordedBy: Qaiser Khan; individualCount: 14; occurrenceID: 95DEA327-AF50-5D1D-A463-FD263E37EB2D; **Taxon:** scientificName: Eupeodescorollae (Fabricius, 1794); order: Diptera; family: Syrphidae; genus: Eupeodes; specificEpithet: corollae; scientificNameAuthorship: (Fabricius, 1794); **Location:** country: Pakistan; countryCode: PK; stateProvince: Balochistan; county: Ziatar District; municipality: Ziarat; decimalLatitude: 30.400139; decimalLongitude: 67.726806; geodeticDatum: WGS84; **Identification:** identifiedBy: Qaiser Khan; **Event:** eventDate: 2019-06-30
- **Type status:** Other material. **Occurrence:** recordedBy: Qaiser Khan; individualCount: 31; occurrenceID: 2F4504B5-5AAF-5747-A9AE-5D77A5538F92; **Taxon:** scientificName: Eupeodescorollae (Fabricius, 1794); order: Diptera; family: Syrphidae; genus: Eupeodes; specificEpithet: corollae; scientificNameAuthorship: (Fabricius, 1794); **Location:** country: Pakistan; countryCode: PK; stateProvince: Balochistan; county: Ziatar District; municipality: Ziarat; decimalLatitude: 30.400139; decimalLongitude: 67.726806; geodeticDatum: WGS84; **Identification:** identifiedBy: Qaiser Khan; **Event:** eventDate: 2019-07-15
- **Type status:** Other material. **Occurrence:** recordedBy: Qaiser Khan; individualCount: 2; occurrenceID: 13F34CE5-E926-547A-872E-18BDBC3849AE; **Taxon:** scientificName: Eupeodescorollae (Fabricius, 1794); order: Diptera; family: Syrphidae; genus: Eupeodes; specificEpithet: corollae; scientificNameAuthorship: (Fabricius, 1794); **Location:** country: Pakistan; countryCode: PK; stateProvince: Balochistan; county: Ziatar District; municipality: Ziarat; decimalLatitude: 30.400139; decimalLongitude: 67.726806; geodeticDatum: WGS84; **Identification:** identifiedBy: Qaiser Khan; **Event:** eventDate: 2019-07-22
- **Type status:** Other material. **Occurrence:** recordedBy: Qaiser Khan; individualCount: 1; occurrenceID: B4ADBB7C-FFE2-5072-90B2-662E175630E8; **Taxon:** scientificName: Eupeodescorollae (Fabricius, 1794); order: Diptera; family: Syrphidae; genus: Eupeodes; specificEpithet: corollae; scientificNameAuthorship: (Fabricius, 1794); **Location:** country: Pakistan; countryCode: PK; stateProvince: Balochistan; county: Ziatar District; municipality: Ziarat; decimalLatitude: 30.400139; decimalLongitude: 67.726806; geodeticDatum: WGS84; **Identification:** identifiedBy: Qaiser Khan; **Event:** eventDate: 2019-07-29
- **Type status:** Other material. **Occurrence:** recordedBy: Qaiser Khan; individualCount: 2; occurrenceID: CED8B222-520A-5DC9-8A7E-9C7C2BB9618F; **Taxon:** scientificName: Eupeodescorollae (Fabricius, 1794); order: Diptera; family: Syrphidae; genus: Eupeodes; specificEpithet: corollae; scientificNameAuthorship: (Fabricius, 1794); **Location:** country: Pakistan; countryCode: PK; stateProvince: Balochistan; county: Ziatar District; municipality: Ziarat; decimalLatitude: 30.400139; decimalLongitude: 67.726806; geodeticDatum: WGS84; **Identification:** identifiedBy: Qaiser Khan; **Event:** eventDate: 2019-08-12
- **Type status:** Other material. **Occurrence:** recordedBy: Qaiser Khan; individualCount: 9; occurrenceID: D5F533E3-A89F-501D-8432-0394156B0859; **Taxon:** scientificName: Eupeodescorollae (Fabricius, 1794); order: Diptera; family: Syrphidae; genus: Eupeodes; specificEpithet: corollae; scientificNameAuthorship: (Fabricius, 1794); **Location:** country: Pakistan; countryCode: PK; stateProvince: Balochistan; county: Ziatar District; municipality: Ziarat; decimalLatitude: 30.400139; decimalLongitude: 67.726806; geodeticDatum: WGS84; **Identification:** identifiedBy: Qaiser Khan; **Event:** eventDate: 2019-08-19

#### Distribution

The species has been documented from all provinces of Pakistan, except Sindh (Shehzad et al. 2017, Turk et al. 2015). Distribution of the species has been reported in the Palearctic Range to the Mediterranean asin, from the coastal states of Africa, down to South Africa as well as in Asia (Tenhumberg and Poehling 1995, Ghorpadé 2015).

#### Notes

**Figure 6.**
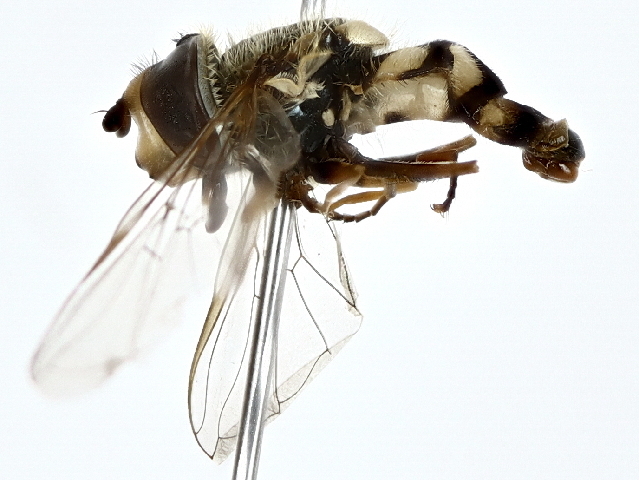
*Eupeodescorolla* (Linnaeus, 1758).

The species has been identified as a potential control agent against aphids and promoting pollination (Anonymous 2004). Its larvae feed on aphids and other scale insects (Rossi et al. 2006). Its presence in the study areas was documented as occurring from April to August and it was first documented in the Juniper ecosystem of Ziarat (Fig. 6).

### Eristalis tenax

(Linnaeus, 1758)

C3BEFF79-B4EF-58B4-907C-233D1EBF6DF3

OR685688

#### Materials

- **Type status:** Other material. **Occurrence:** recordedBy: Qaiser Khan; individualCount: 6; occurrenceID: 77E212BC-C9FC-5195-A54E-07A792FE1CDB; **Taxon:** scientificName: Eristalistenax (Linnaeus, 1758); order: Diptera; family: Syrphidae; genus: Eristalis; specificEpithet: tenax; scientificNameAuthorship: (Linnaeus, 1758); **Location:** country: Pakistan; countryCode: PK; stateProvince: Balochistan; county: Ziatar District; municipality: Ziarat; decimalLatitude: 30.400139; decimalLongitude: 67.726806; geodeticDatum: WGS84; **Identification:** identifiedBy: Qaiser Khan; **Event:** eventDate: 2019-04-15
- **Type status:** Other material. **Occurrence:** recordedBy: Qaiser Khan; individualCount: 13; occurrenceID: 702EC7F6-ADE6-5838-BCE4-5175828A0B69; **Taxon:** scientificName: Eristalistenax (Linnaeus, 1758); order: Diptera; family: Syrphidae; genus: Eristalis; specificEpithet: tenax; scientificNameAuthorship: (Linnaeus, 1758); **Location:** country: Pakistan; countryCode: PK; stateProvince: Balochistan; county: Ziatar District; municipality: Ziarat; decimalLatitude: 30.400139; decimalLongitude: 67.726806; geodeticDatum: WGS84; **Identification:** identifiedBy: Qaiser Khan; **Event:** eventDate: 2019-04-22
- **Type status:** Other material. **Occurrence:** recordedBy: Qaiser Khan; individualCount: 24; occurrenceID: 3FB170CE-AA09-54EC-BB3D-8D8FF7B9C9EB; **Taxon:** scientificName: Eristalistenax (Linnaeus, 1758); order: Diptera; family: Syrphidae; genus: Eristalis; specificEpithet: tenax; scientificNameAuthorship: (Linnaeus, 1758); **Location:** country: Pakistan; countryCode: PK; stateProvince: Balochistan; county: Ziatar District; municipality: Ziarat; decimalLatitude: 30.400139; decimalLongitude: 67.726806; geodeticDatum: WGS84; **Identification:** identifiedBy: Qaiser Khan; **Event:** eventDate: 2019-05-13
- **Type status:** Other material. **Occurrence:** recordedBy: Qaiser Khan; individualCount: 3; occurrenceID: E4ACF264-2B2F-51AF-8EEE-63F11CCD3B4D; **Taxon:** scientificName: Eristalistenax (Linnaeus, 1758); order: Diptera; family: Syrphidae; genus: Eristalis; specificEpithet: tenax; scientificNameAuthorship: (Linnaeus, 1758); **Location:** country: Pakistan; countryCode: PK; stateProvince: Balochistan; county: Ziatar District; municipality: Ziarat; decimalLatitude: 30.400139; decimalLongitude: 67.726806; geodeticDatum: WGS84; **Identification:** identifiedBy: Qaiser Khan; **Event:** eventDate: 2019-05-27
- **Type status:** Other material. **Occurrence:** recordedBy: Qaiser Khan; individualCount: 6; occurrenceID: 8A030134-7FBE-5176-B899-E5588F177D50; **Taxon:** scientificName: Eristalistenax (Linnaeus, 1758); order: Diptera; family: Syrphidae; genus: Eristalis; specificEpithet: tenax; scientificNameAuthorship: (Linnaeus, 1758); **Location:** country: Pakistan; countryCode: PK; stateProvince: Balochistan; county: Ziatar District; municipality: Ziarat; decimalLatitude: 30.400139; decimalLongitude: 67.726806; geodeticDatum: WGS84; **Identification:** identifiedBy: Qaiser Khan; **Event:** eventDate: 3019-06-30

#### Distribution

Except for the Sindh Province, where it was first recorded, this species is widely distributed in Pakistan (Ghorpadé and Shehzad 2013, Arif et al. 2013). Furthermore, it has global distribution (Catts and Mullen 2002, Howlett and Gee 2019) except Antarctica (Thompson and Rotheray 1998).

#### Notes

**Figure 7.**
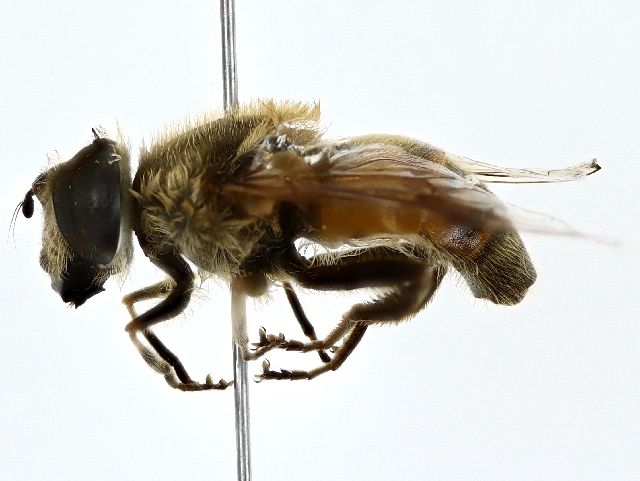
*Eristalistenax* (Linnaeus, 1758).

Cosmopolitan, this species was observed from April to late June in Ziarat (Fig. 7).

### Gonia ornata

Meigen, 1826

AE0A3270-C8AE-5136-B89A-D7EAFF3D43BE

OR685691

#### Materials

- **Type status:** Other material. **Occurrence:** recordedBy: Qaiser Khan; individualCount: 2; occurrenceID: D0F355B1-9A37-540F-827F-B2A96815C9DB; **Taxon:** scientificName: Goniaornata Meigen, 1826; order: Diptera; family: Tachinidae; genus: Gonia; specificEpithet: ornata; scientificNameAuthorship: Meigen, 1826; **Location:** country: Pakistan; countryCode: PK; stateProvince: Balochistan; county: Ziatar District; municipality: Ziarat; decimalLatitude: 30.400139; decimalLongitude: 67.726806; geodeticDatum: WGS84; **Identification:** identifiedBy: Qaiser Khan; **Event:** eventDate: 2019-01-21
- **Type status:** Other material. **Occurrence:** recordedBy: Qaiser Khan; individualCount: 1; occurrenceID: 2A978DE2-08D1-5E52-A6D1-EA698C96C362; **Taxon:** scientificName: Goniaornata Meigen, 1826; order: Diptera; family: Tachinidae; genus: Gonia; specificEpithet: ornata; scientificNameAuthorship: Meigen, 1826; **Location:** country: Pakistan; countryCode: PK; stateProvince: Balochistan; county: Ziatar District; municipality: Ziarat; decimalLatitude: 30.400139; decimalLongitude: 67.726806; geodeticDatum: WGS84; **Identification:** identifiedBy: Qaiser Khan; **Event:** eventDate: 2019-09-09
- **Type status:** Other material. **Occurrence:** recordedBy: Qaiser Khan; individualCount: 11; occurrenceID: BF5805B2-6862-59E2-B9F2-969C421124D4; **Taxon:** scientificName: Goniaornata Meigen, 1826; order: Diptera; family: Tachinidae; genus: Gonia; specificEpithet: ornata; scientificNameAuthorship: Meigen, 1826; **Location:** country: Pakistan; countryCode: PK; stateProvince: Balochistan; county: Ziatar District; municipality: Ziarat; decimalLatitude: 30.400139; decimalLongitude: 67.726806; geodeticDatum: WGS84; **Identification:** identifiedBy: Qaiser Khan; **Event:** eventDate: 2019-10-07
- **Type status:** Other material. **Occurrence:** recordedBy: Qaiser Khan; individualCount: 17; occurrenceID: 60D84F9D-E03A-503D-A710-2ACFE23D1B7A; **Taxon:** scientificName: Goniaornata Meigen, 1826; order: Diptera; family: Tachinidae; genus: Gonia; specificEpithet: ornata; scientificNameAuthorship: Meigen, 1826; **Location:** country: Pakistan; countryCode: PK; stateProvince: Balochistan; county: Ziatar District; municipality: Ziarat; decimalLatitude: 30.400139; decimalLongitude: 67.726806; geodeticDatum: WGS84; **Identification:** identifiedBy: Qaiser Khan; **Event:** eventDate: 2019-11-18
- **Type status:** Other material. **Occurrence:** recordedBy: Qaiser Khan; individualCount: 7; occurrenceID: 2E9FBBAB-0B65-50A4-81CF-60FDC81E5107; **Taxon:** scientificName: Goniaornata Meigen, 1826; order: Diptera; family: Tachinidae; genus: Gonia; specificEpithet: ornata; scientificNameAuthorship: Meigen, 1826; **Location:** country: Pakistan; countryCode: PK; stateProvince: Balochistan; county: Ziatar District; municipality: Ziarat; decimalLatitude: 30.400139; decimalLongitude: 67.726806; geodeticDatum: WGS84; **Identification:** identifiedBy: Qaiser Khan; **Event:** eventDate: 2019-12-16
- **Type status:** Other material. **Occurrence:** recordedBy: Qaiser Khan; individualCount: 2; occurrenceID: 194D9792-559A-5D8A-B437-2E7FE4ADF580; **Taxon:** scientificName: Goniaornata Meigen, 1826; order: Diptera; family: Tachinidae; genus: Gonia; specificEpithet: ornata; scientificNameAuthorship: Meigen, 1826; **Location:** country: Pakistan; countryCode: PK; stateProvince: Balochistan; county: Ziatar District; municipality: Ziarat; decimalLatitude: 30.400139; decimalLongitude: 67.726806; geodeticDatum: WGS84; **Identification:** identifiedBy: Qaiser Khan; **Event:** eventDate: 2019-12-24
- **Type status:** Other material. **Occurrence:** recordedBy: Qaiser Khan; individualCount: 3; occurrenceID: 2E90723D-2FA7-5EA0-B5DE-74291CB14F52; **Taxon:** scientificName: Goniaornata Meigen, 1826; order: Diptera; family: Tachinidae; genus: Gonia; specificEpithet: ornata; scientificNameAuthorship: Meigen, 1826; **Location:** country: Pakistan; countryCode: PK; stateProvince: Balochistan; county: Ziatar District; municipality: Ziarat; decimalLatitude: 30.400139; decimalLongitude: 67.726806; geodeticDatum: WGS84; **Identification:** identifiedBy: Qaiser Khan; **Event:** eventDate: 2019-12-31

#### Distribution

This species is new for the fauna of Pakistan. Its has been reported from China, Europe, Middle Eastern countries, Mongolia, Russia, Transcaucasia and Asia (Hou et al. 2018, Lee and Han 2010, Tschorsnig and Herting 1994).

#### Notes

**Figure 8.**
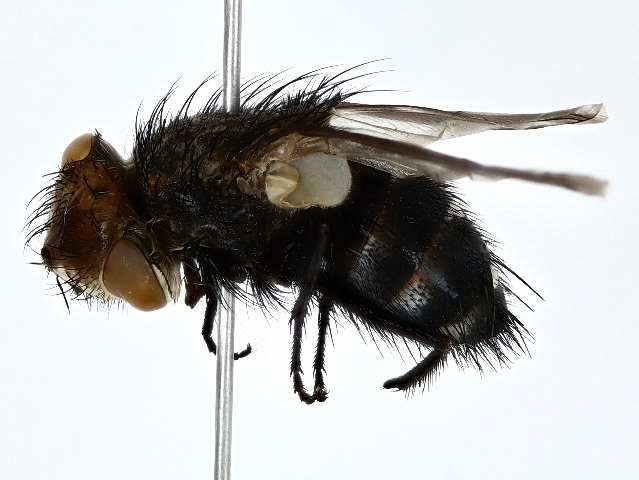
*Goniaornata* (Meigen, 1826).

*Gonia* is an important agent for pest control in agriculture and forestry (Morrison 1940). The species was observed from October to January (Fig. 8).

### Lucilia sericata

(Meigen, 1826)

084889ED-1735-562C-8882-42813EE1A533

OR685692

#### Materials

- **Type status:** Other material. **Occurrence:** recordedBy: Qaiser Khan; individualCount: 2; occurrenceID: 612955E1-3D06-5039-BA2A-F8189D0CFC32; **Taxon:** scientificName: *Luciliasericata* (Meigen, 1826); order: Diptera; family: Calliphoridae; **Location:** country: Pakistan; countryCode: PK; stateProvince: Balochistan; county: Ziarat Distric; municipality: Ziarat; decimalLatitude: 30.400139; decimalLongitude: 67.726806; geodeticDatum: WGS8; **Identification:** identifiedBy: Qaiser Khan; **Event:** eventDate: 2019-04-22
- **Type status:** Other material. **Occurrence:** recordedBy: Qaiser Khan; individualCount: 2; occurrenceID: 612955E1-3D06-5039-BA2A-F8189D0CFC32; **Taxon:** scientificName: *Luciliasericata* (Meigen, 1826); order: Diptera; family: Calliphoridae; **Location:** country: Pakistan; countryCode: PK; stateProvince: Balochistan; county: Ziarat Distric; municipality: Ziarat; decimalLatitude: 30.400139; decimalLongitude: 67.726806; geodeticDatum: WGS8; **Identification:** identifiedBy: Qaiser Khan; **Event:** eventDate: 2019-05-20
- **Type status:** Other material. **Occurrence:** recordedBy: Qaiser Khan; individualCount: 1; occurrenceID: 612955E1-3D06-5039-BA2A-F8189D0CFC32; **Taxon:** scientificName: *Luciliasericata* (Meigen, 1826); order: Diptera; family: Calliphoridae; **Location:** country: Pakistan; countryCode: PK; stateProvince: Balochistan; county: Ziarat Distric; municipality: Ziarat; decimalLatitude: 30.400139; decimalLongitude: 67.726806; geodeticDatum: WGS8; **Identification:** identifiedBy: Qaiser Khan; **Event:** eventDate: 2019-05-27
- **Type status:** Other material. **Occurrence:** recordedBy: Qaiser Khan; individualCount: 3; occurrenceID: 612955E1-3D06-5039-BA2A-F8189D0CFC32; **Taxon:** scientificName: *Luciliasericata* (Meigen, 1826); order: Diptera; family: Calliphoridae; **Location:** country: Pakistan; countryCode: PK; stateProvince: Balochistan; county: Ziarat Distric; municipality: Ziarat; decimalLatitude: 30.400139; decimalLongitude: 67.726806; geodeticDatum: WGS8; **Identification:** identifiedBy: Qaiser Khan; **Event:** eventDate: 2019-06-10
- **Type status:** Other material. **Occurrence:** recordedBy: Qaiser Khan; individualCount: 1; occurrenceID: 612955E1-3D06-5039-BA2A-F8189D0CFC32; **Taxon:** scientificName: *Luciliasericata* (Meigen, 1826); order: Diptera; family: Calliphoridae; **Location:** country: Pakistan; countryCode: PK; stateProvince: Balochistan; county: Ziarat Distric; municipality: Ziarat; decimalLatitude: 30.400139; decimalLongitude: 67.726806; geodeticDatum: WGS8; **Identification:** identifiedBy: Qaiser Khan; **Event:** eventDate: 2019-07-08
- **Type status:** Other material. **Occurrence:** recordedBy: Qaiser Khan; individualCount: 1; occurrenceID: 612955E1-3D06-5039-BA2A-F8189D0CFC32; **Taxon:** scientificName: *Luciliasericata* (Meigen, 1826); order: Diptera; family: Calliphoridae; **Location:** country: Pakistan; countryCode: PK; stateProvince: Balochistan; county: Ziarat Distric; municipality: Ziarat; decimalLatitude: 30.400139; decimalLongitude: 67.726806; geodeticDatum: WGS8; **Identification:** identifiedBy: Qaiser Khan; **Event:** eventDate: 2019-07-15
- **Type status:** Other material. **Occurrence:** recordedBy: Qaiser Khan; individualCount: 1; occurrenceID: 612955E1-3D06-5039-BA2A-F8189D0CFC32; **Taxon:** scientificName: *Luciliasericata* (Meigen, 1826); order: Diptera; family: Calliphoridae; **Location:** country: Pakistan; countryCode: PK; stateProvince: Balochistan; county: Ziarat Distric; municipality: Ziarat; decimalLatitude: 30.400139; decimalLongitude: 67.726806; geodeticDatum: WGS8; **Identification:** identifiedBy: Qaiser Khan; **Event:** eventDate: 2019-07-29
- **Type status:** Other material. **Occurrence:** recordedBy: Qaiser Khan; individualCount: 6; occurrenceID: 612955E1-3D06-5039-BA2A-F8189D0CFC32; **Taxon:** scientificName: *Luciliasericata* (Meigen, 1826); order: Diptera; family: Calliphoridae; **Location:** country: Pakistan; countryCode: PK; stateProvince: Balochistan; county: Ziarat Distric; municipality: Ziarat; decimalLatitude: 30.400139; decimalLongitude: 67.726806; geodeticDatum: WGS8; **Identification:** identifiedBy: Qaiser Khan; **Event:** eventDate: 2019-08-26
- **Type status:** Other material. **Occurrence:** recordedBy: Qaiser Khan; individualCount: 14; occurrenceID: 612955E1-3D06-5039-BA2A-F8189D0CFC32; **Taxon:** scientificName: *Luciliasericata* (Meigen, 1826); order: Diptera; family: Calliphoridae; **Location:** country: Pakistan; countryCode: PK; stateProvince: Balochistan; county: Ziarat Distric; municipality: Ziarat; decimalLatitude: 30.400139; decimalLongitude: 67.726806; geodeticDatum: WGS8; **Identification:** identifiedBy: Qaiser Khan; **Event:** eventDate: 2019-09-09
- **Type status:** Other material. **Occurrence:** recordedBy: Qaiser Khan; individualCount: 21; occurrenceID: 612955E1-3D06-5039-BA2A-F8189D0CFC32; **Taxon:** scientificName: *Luciliasericata* (Meigen, 1826); order: Diptera; family: Calliphoridae; **Location:** country: Pakistan; countryCode: PK; stateProvince: Balochistan; county: Ziarat Distric; municipality: Ziarat; decimalLatitude: 30.400139; decimalLongitude: 67.726806; geodeticDatum: WGS8; **Identification:** identifiedBy: Qaiser Khan; **Event:** eventDate: 2019-10-07
- **Type status:** Other material. **Occurrence:** recordedBy: Qaiser Khan; individualCount: 1; occurrenceID: 612955E1-3D06-5039-BA2A-F8189D0CFC32; **Taxon:** scientificName: *Luciliasericata* (Meigen, 1826); order: Diptera; family: Calliphoridae; **Location:** country: Pakistan; countryCode: PK; stateProvince: Balochistan; county: Ziarat Distric; municipality: Ziarat; decimalLatitude: 30.400139; decimalLongitude: 67.726806; geodeticDatum: WGS8; **Identification:** identifiedBy: Qaiser Khan; **Event:** eventDate: 2019-10-14
- **Type status:** Other material. **Occurrence:** recordedBy: Qaiser Khan; individualCount: 31; occurrenceID: 612955E1-3D06-5039-BA2A-F8189D0CFC32; **Taxon:** scientificName: *Luciliasericata* (Meigen, 1826); order: Diptera; family: Calliphoridae; **Location:** country: Pakistan; countryCode: PK; stateProvince: Balochistan; county: Ziarat Distric; municipality: Ziarat; decimalLatitude: 30.400139; decimalLongitude: 67.726806; geodeticDatum: WGS8; **Identification:** identifiedBy: Qaiser Khan; **Event:** eventDate: 2019-11-18
- **Type status:** Other material. **Occurrence:** recordedBy: Qaiser Khan; individualCount: 3; occurrenceID: 612955E1-3D06-5039-BA2A-F8189D0CFC32; **Taxon:** scientificName: *Luciliasericata* (Meigen, 1826); order: Diptera; family: Calliphoridae; **Location:** country: Pakistan; countryCode: PK; stateProvince: Balochistan; county: Ziarat Distric; municipality: Ziarat; decimalLatitude: 30.400139; decimalLongitude: 67.726806; geodeticDatum: WGS8; **Identification:** identifiedBy: Qaiser Khan; **Event:** eventDate: 2018-12-09

#### Distribution

Species widely distributed in the Middle East and Southeast Asia (China, Japan, Korea, Pakistan, Philippines, Sri Lanka and Taiwan); it was also reported from Europe, the United States and outhern Canada (Kurahashi and Afzal 2002, Akbarzadeh et al. 2015, Verves and Khrokalo 2010).

#### Notes

**Figure 9.**
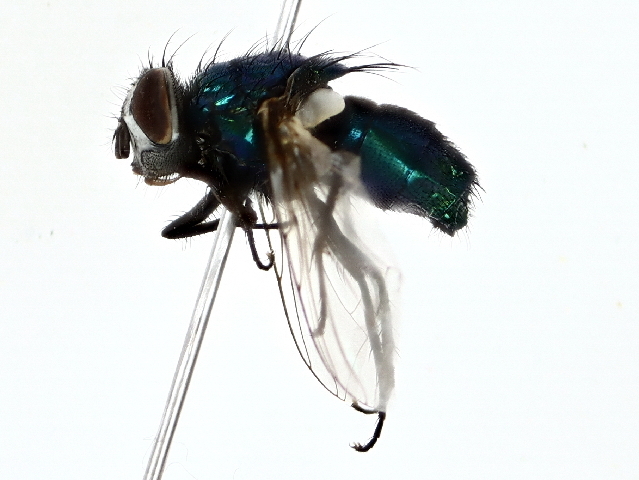
*Luciliasericata* (Meigen, 1826).

In the studied area, this species was observed to be active from April to December (Fig. 9).

### Paragus quadrifasciatus

Meigen, 1822

793BCD02-B5AB-5B83-8EE1-A6D011F5E797

OR685693

#### Materials

- **Type status:** Other material. **Occurrence:** recordedBy: Qaiser Khan; individualCount: 2; occurrenceID: 331053E6-6FE0-5595-9EFE-1F13F400E3C7; **Taxon:** scientificName: Paragusquadrifasciatus Meigen, 1822; order: Diptera; family: Syrphidae; genus: Paragus; specificEpithet: quadrifasciatus; scientificNameAuthorship: Meigen, 1822; **Location:** country: Pakistan; countryCode: PK; stateProvince: Balochistan; county: Ziatar District; municipality: Ziarat; decimalLatitude: 30.400139; decimalLongitude: 67.726806; geodeticDatum: WGS84; **Identification:** identifiedBy: Qaiser Khan; **Event:** eventDate: 2019-03-04
- **Type status:** Other material. **Occurrence:** recordedBy: Qaiser Khan; individualCount: 102; occurrenceID: 96DBE415-B9BE-5158-9162-4DC644795DAE; **Taxon:** scientificName: Paragusquadrifasciatus Meigen, 1822; order: Diptera; family: Syrphidae; genus: Paragus; specificEpithet: quadrifasciatus; scientificNameAuthorship: Meigen, 1822; **Location:** country: Pakistan; countryCode: PK; stateProvince: Balochistan; county: Ziatar District; municipality: Ziarat; decimalLatitude: 30.400139; decimalLongitude: 67.726806; geodeticDatum: WGS84; **Identification:** identifiedBy: Qaiser Khan; **Event:** eventDate: 2019-04-08
- **Type status:** Other material. **Occurrence:** recordedBy: Qaiser Khan; individualCount: 45; occurrenceID: 7458E0CC-2450-5749-A385-EF8AA2E789BB; **Taxon:** scientificName: Paragusquadrifasciatus Meigen, 1822; order: Diptera; family: Syrphidae; genus: Paragus; specificEpithet: quadrifasciatus; scientificNameAuthorship: Meigen, 1822; **Location:** country: Pakistan; countryCode: PK; stateProvince: Balochistan; county: Ziatar District; municipality: Ziarat; decimalLatitude: 30.400139; decimalLongitude: 67.726806; geodeticDatum: WGS84; **Identification:** identifiedBy: Qaiser Khan; **Event:** eventDate: 2019-04-15
- **Type status:** Other material. **Occurrence:** recordedBy: Qaiser Khan; individualCount: 11; occurrenceID: CDB89A0C-148C-52AF-B7CC-B9E325B46EAE; **Taxon:** scientificName: Paragusquadrifasciatus Meigen, 1822; order: Diptera; family: Syrphidae; genus: Paragus; specificEpithet: quadrifasciatus; scientificNameAuthorship: Meigen, 1822; **Location:** country: Pakistan; countryCode: PK; stateProvince: Balochistan; county: Ziatar District; municipality: Ziarat; decimalLatitude: 30.400139; decimalLongitude: 67.726806; geodeticDatum: WGS84; **Identification:** identifiedBy: Qaiser Khan; **Event:** eventDate: 2019-04-22
- **Type status:** Other material. **Occurrence:** recordedBy: Qaiser Khan; individualCount: 72; occurrenceID: 52EAF04E-9DD2-5E66-9323-4C505C69DAF7; **Taxon:** scientificName: Paragusquadrifasciatus Meigen, 1822; order: Diptera; family: Syrphidae; genus: Paragus; specificEpithet: quadrifasciatus; scientificNameAuthorship: Meigen, 1822; **Location:** country: Pakistan; countryCode: PK; stateProvince: Balochistan; county: Ziatar District; municipality: Ziarat; decimalLatitude: 30.400139; decimalLongitude: 67.726806; geodeticDatum: WGS84; **Identification:** identifiedBy: Qaiser Khan; **Event:** eventDate: 2019-04-29
- **Type status:** Other material. **Occurrence:** recordedBy: Qaiser Khan; individualCount: 61; occurrenceID: 30295DB6-5D89-57E0-B7CD-66F9ABFA805D; **Taxon:** scientificName: Paragusquadrifasciatus Meigen, 1822; order: Diptera; family: Syrphidae; genus: Paragus; specificEpithet: quadrifasciatus; scientificNameAuthorship: Meigen, 1822; **Location:** country: Pakistan; countryCode: PK; stateProvince: Balochistan; county: Ziatar District; municipality: Ziarat; decimalLatitude: 30.400139; decimalLongitude: 67.726806; geodeticDatum: WGS84; **Identification:** identifiedBy: Qaiser Khan; **Event:** eventDate: 2019-05-13
- **Type status:** Other material. **Occurrence:** recordedBy: Qaiser Khan; individualCount: 32; occurrenceID: ADBB4567-C3D8-515E-9783-B08FF7CC3450; **Taxon:** scientificName: Paragusquadrifasciatus Meigen, 1822; order: Diptera; family: Syrphidae; genus: Paragus; specificEpithet: quadrifasciatus; scientificNameAuthorship: Meigen, 1822; **Location:** country: Pakistan; countryCode: PK; stateProvince: Balochistan; county: Ziatar District; municipality: Ziarat; decimalLatitude: 30.400139; decimalLongitude: 67.726806; geodeticDatum: WGS84; **Identification:** identifiedBy: Qaiser Khan; **Event:** eventDate: 2019-05-20
- **Type status:** Other material. **Occurrence:** recordedBy: Qaiser Khan; individualCount: 12; occurrenceID: AADE9817-6015-518D-8B9F-7194C40BB72F; **Taxon:** scientificName: Paragusquadrifasciatus Meigen, 1822; order: Diptera; family: Syrphidae; genus: Paragus; specificEpithet: quadrifasciatus; scientificNameAuthorship: Meigen, 1822; **Location:** country: Pakistan; countryCode: PK; stateProvince: Balochistan; county: Ziatar District; municipality: Ziarat; decimalLatitude: 30.400139; decimalLongitude: 67.726806; geodeticDatum: WGS84; **Identification:** identifiedBy: Qaiser Khan; **Event:** eventDate: 2019-05-27
- **Type status:** Other material. **Occurrence:** recordedBy: Qaiser Khan; individualCount: 10; occurrenceID: 3E80ABD5-BE78-5EC1-8B39-8F4DB5E8B681; **Taxon:** scientificName: Paragusquadrifasciatus Meigen, 1822; order: Diptera; family: Syrphidae; genus: Paragus; specificEpithet: quadrifasciatus; scientificNameAuthorship: Meigen, 1822; **Location:** country: Pakistan; countryCode: PK; stateProvince: Balochistan; county: Ziatar District; municipality: Ziarat; decimalLatitude: 30.400139; decimalLongitude: 67.726806; geodeticDatum: WGS84; **Identification:** identifiedBy: Qaiser Khan; **Event:** eventDate: 2019-06-17
- **Type status:** Other material. **Occurrence:** recordedBy: Qaiser Khan; individualCount: 13; occurrenceID: DE873EC5-953A-5954-AF29-F3802CA565C7; **Taxon:** scientificName: Paragusquadrifasciatus Meigen, 1822; order: Diptera; family: Syrphidae; genus: Paragus; specificEpithet: quadrifasciatus; scientificNameAuthorship: Meigen, 1822; **Location:** country: Pakistan; countryCode: PK; stateProvince: Balochistan; county: Ziatar District; municipality: Ziarat; decimalLatitude: 30.400139; decimalLongitude: 67.726806; geodeticDatum: WGS84; **Identification:** identifiedBy: Qaiser Khan; **Event:** eventDate: 2019-06-24

#### Distribution

Recorded in Afghanistan, China, India, Iran, Kazakhstan, Kirghizia, Pakistan, Russia, Tajikistan and Turkmenistan (Ghorpadé and Shehzad 2013, Ghorpadé 2015).

#### Notes

**Figure 10.**
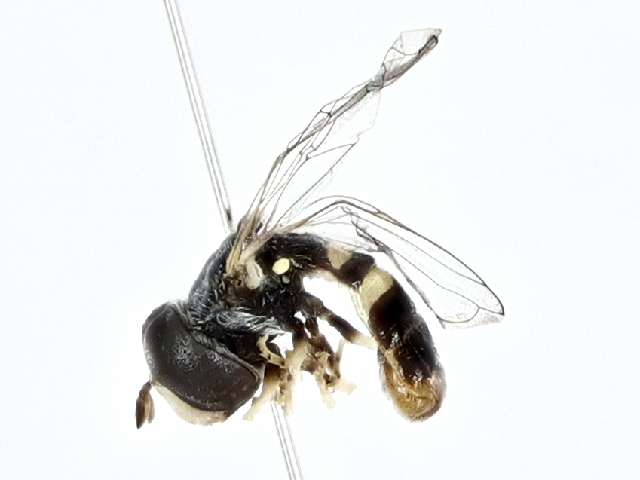
*Paragusquadrifasciatus* (Linnaeus,1758).

Its presence serves as an important component of aphid control (Krsteska 2010). This newlydocumented species of aphid within the Juniper ecosystem of Ziarat is a vital part of the natural agroecosystem. It was observed between April and July (Fig. 10).

### Pollenia rudis

(Fabricius, 1794)

91B60E32-E903-5975-919C-07D100B585B5

OR685694

OR685695

#### Materials

- **Type status:** Other material. **Occurrence:** recordedBy: Qaiser Khan; individualCount: 8; occurrenceID: CD963EB4-53E3-5370-8171-BA7629D2AC94; **Taxon:** scientificName: Polleniarudis (Fabricius, 1794); order: Diptera; family: Polleniidae; genus: Pollenia; specificEpithet: rudis; scientificNameAuthorship: (Fabricius, 1794); **Location:** country: Pakistan; countryCode: PK; stateProvince: Balochistan; county: Ziatar District; municipality: Ziarat; decimalLatitude: 30.400139; decimalLongitude: 67.726806; geodeticDatum: WGS84; **Identification:** identifiedBy: Qaiser Khan; **Event:** eventDate: 2019-06-24
- **Type status:** Other material. **Occurrence:** recordedBy: Qaiser Khan; individualCount: 12; occurrenceID: F62CEF4C-0F02-5374-907F-32E751689B21; **Taxon:** scientificName: Polleniarudis (Fabricius, 1794); order: Diptera; family: Polleniidae; genus: Pollenia; specificEpithet: rudis; scientificNameAuthorship: (Fabricius, 1794); **Location:** country: Pakistan; countryCode: PK; stateProvince: Balochistan; county: Ziatar District; municipality: Ziarat; decimalLatitude: 30.400139; decimalLongitude: 67.726806; geodeticDatum: WGS84; **Identification:** identifiedBy: Qaiser Khan; **Event:** eventDate: 2019-07-22
- **Type status:** Other material. **Occurrence:** recordedBy: Qaiser Khan; individualCount: 10; occurrenceID: 93CEB988-CEB5-5A1B-953D-A1BA57E0A3AB; **Taxon:** scientificName: Polleniarudis (Fabricius, 1794); order: Diptera; family: Polleniidae; genus: Pollenia; specificEpithet: rudis; scientificNameAuthorship: (Fabricius, 1794); **Location:** country: Pakistan; countryCode: PK; stateProvince: Balochistan; county: Ziatar District; municipality: Ziarat; decimalLatitude: 30.400139; decimalLongitude: 67.726806; geodeticDatum: WGS84; **Identification:** identifiedBy: Qaiser Khan; **Event:** eventDate: 2019-08-26
- **Type status:** Other material. **Occurrence:** recordedBy: Qaiser Khan; individualCount: 13; occurrenceID: E0FBFF7E-195C-5A1B-8DBD-08E4FE633B42; **Taxon:** scientificName: Polleniarudis (Fabricius, 1794); order: Diptera; family: Polleniidae; genus: Pollenia; specificEpithet: rudis; scientificNameAuthorship: (Fabricius, 1794); **Location:** country: Pakistan; countryCode: PK; stateProvince: Balochistan; county: Ziatar District; municipality: Ziarat; decimalLatitude: 30.400139; decimalLongitude: 67.726806; geodeticDatum: WGS84; **Identification:** identifiedBy: Qaiser Khan; **Event:** eventDate: 2019-09-09
- **Type status:** Other material. **Occurrence:** recordedBy: Qaiser Khan; individualCount: 1; occurrenceID: C5CCF762-8E6D-5A1E-9BBF-9AFAC934492F; **Taxon:** scientificName: Polleniarudis (Fabricius, 1794); order: Diptera; family: Polleniidae; genus: Pollenia; specificEpithet: rudis; scientificNameAuthorship: (Fabricius, 1794); **Location:** country: Pakistan; countryCode: PK; stateProvince: Balochistan; county: Ziatar District; municipality: Ziarat; decimalLatitude: 30.400139; decimalLongitude: 67.726806; geodeticDatum: WGS84; **Identification:** identifiedBy: Qaiser Khan; **Event:** eventDate: 2019-09-16
- **Type status:** Other material. **Occurrence:** recordedBy: Qaiser Khan; individualCount: 3; occurrenceID: 80880189-4F6A-5626-86FB-36F4BDF40430; **Taxon:** scientificName: Polleniarudis (Fabricius, 1794); order: Diptera; family: Polleniidae; genus: Pollenia; specificEpithet: rudis; scientificNameAuthorship: (Fabricius, 1794); **Location:** country: Pakistan; countryCode: PK; stateProvince: Balochistan; county: Ziatar District; municipality: Ziarat; decimalLatitude: 30.400139; decimalLongitude: 67.726806; geodeticDatum: WGS84; **Identification:** identifiedBy: Qaiser Khan; **Event:** eventDate: 2019-10-14

#### Distribution

It has been reported from all provinces of Pakistan except Sindh (Ghorpadé 2015, Ghorpadé and Shehzad 2013). The species is cosmopolitan (Byrd and Castner 2010, Chiewchanvit et al. 2017).

#### Notes

**Figure 11.**
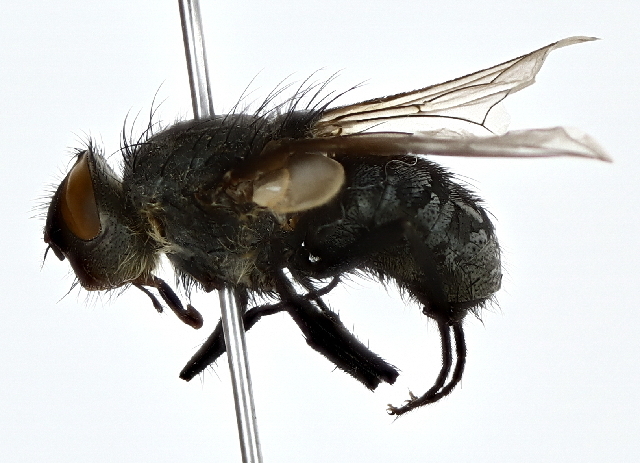
*Polleniarudis* (Fabricius, 1794).

It is commonly known as cluster fly and these are common household nuisances during autumn and winter months. They are parasitic to earthworms (Capinera 2008). Adults are found during June to October in the study area. This is the first record for the Province (Fig. 11).

### Ravinia pernix

(Harris, 1780)

9373A82D-A7E9-593D-9D93-1AF30DB59FC4

OR685696

#### Materials

- **Type status:** Other material. **Occurrence:** recordedBy: Qaiser Khan; individualCount: 3; occurrenceID: 63932800-54ED-54C4-ADB2-90986719CC34; **Taxon:** scientificName: Raviniapernix (Harris, 1780); order: Diptera; family: Sarcophagidae; genus: Ravinia; specificEpithet: pernix; scientificNameAuthorship: (Harris, 1780); **Location:** country: Pakistan; countryCode: PK; stateProvince: Balochistan; county: Ziatar District; municipality: Ziarat; decimalLatitude: 30.400139; decimalLongitude: 67.726806; geodeticDatum: WGS84; **Identification:** identifiedBy: Qaiser Khan; **Event:** eventDate: 2019-05-13
- **Type status:** Other material. **Occurrence:** recordedBy: Qaiser Khan; individualCount: 1; occurrenceID: 679B0802-B416-54CE-B391-785FF29DCB5A; **Taxon:** scientificName: Raviniapernix (Harris, 1780); order: Diptera; family: Sarcophagidae; genus: Ravinia; specificEpithet: pernix; scientificNameAuthorship: (Harris, 1780); **Location:** country: Pakistan; countryCode: PK; stateProvince: Balochistan; county: Ziatar District; municipality: Ziarat; decimalLatitude: 30.400139; decimalLongitude: 67.726806; geodeticDatum: WGS84; **Identification:** identifiedBy: Qaiser Khan; **Event:** eventDate: 2019-06-24
- **Type status:** Other material. **Occurrence:** recordedBy: Qaiser Khan; individualCount: 9; occurrenceID: 721AC80E-CC47-54E1-B705-8A136566D496; **Taxon:** scientificName: Raviniapernix (Harris, 1780); order: Diptera; family: Sarcophagidae; genus: Ravinia; specificEpithet: pernix; scientificNameAuthorship: (Harris, 1780); **Location:** country: Pakistan; countryCode: PK; stateProvince: Balochistan; county: Ziatar District; municipality: Ziarat; decimalLatitude: 30.400139; decimalLongitude: 67.726806; geodeticDatum: WGS84; **Identification:** identifiedBy: Qaiser Khan; **Event:** eventDate: 2019-06-30
- **Type status:** Other material. **Occurrence:** recordedBy: Qaiser Khan; individualCount: 2; occurrenceID: 9BBB834A-794A-5F20-ACC9-1DD7C2120B62; **Taxon:** scientificName: Raviniapernix (Harris, 1780); order: Diptera; family: Sarcophagidae; genus: Ravinia; specificEpithet: pernix; scientificNameAuthorship: (Harris, 1780); **Location:** country: Pakistan; countryCode: PK; stateProvince: Balochistan; county: Ziatar District; municipality: Ziarat; decimalLatitude: 30.400139; decimalLongitude: 67.726806; geodeticDatum: WGS84; **Identification:** identifiedBy: Qaiser Khan; **Event:** eventDate: 2019-07-15
- **Type status:** Other material. **Occurrence:** recordedBy: Qaiser Khan; individualCount: 11; occurrenceID: AB5D0A34-5120-5F4F-904A-52DD12D686D1; **Taxon:** scientificName: Raviniapernix (Harris, 1780); order: Diptera; family: Sarcophagidae; genus: Ravinia; specificEpithet: pernix; scientificNameAuthorship: (Harris, 1780); **Location:** country: Pakistan; countryCode: PK; stateProvince: Balochistan; county: Ziatar District; municipality: Ziarat; decimalLatitude: 30.400139; decimalLongitude: 67.726806; geodeticDatum: WGS84; **Identification:** identifiedBy: Qaiser Khan; **Event:** eventDate: 2019-07-22
- **Type status:** Other material. **Occurrence:** recordedBy: Qaiser Khan; individualCount: 4; occurrenceID: 2B301EE0-8381-5E13-9329-30C303A2D9D0; **Taxon:** scientificName: Raviniapernix (Harris, 1780); order: Diptera; family: Sarcophagidae; genus: Ravinia; specificEpithet: pernix; scientificNameAuthorship: (Harris, 1780); **Location:** country: Pakistan; countryCode: PK; stateProvince: Balochistan; county: Ziatar District; municipality: Ziarat; decimalLatitude: 30.400139; decimalLongitude: 67.726806; geodeticDatum: WGS84; **Identification:** identifiedBy: Qaiser Khan; **Event:** eventDate: 2019-08-12
- **Type status:** Other material. **Occurrence:** recordedBy: Qaiser Khan; individualCount: 4; occurrenceID: 5D8559F1-75D7-5A9F-A13B-BE8780F11A61; **Taxon:** scientificName: Raviniapernix (Harris, 1780); order: Diptera; family: Sarcophagidae; genus: Ravinia; specificEpithet: pernix; scientificNameAuthorship: (Harris, 1780); **Location:** country: Pakistan; countryCode: PK; stateProvince: Balochistan; county: Ziatar District; municipality: Ziarat; decimalLatitude: 30.400139; decimalLongitude: 67.726806; geodeticDatum: WGS84; **Identification:** identifiedBy: Qaiser Khan; **Event:** eventDate: 2019-08-19
- **Type status:** Other material. **Occurrence:** recordedBy: Qaiser Khan; individualCount: 21; occurrenceID: 7B4E9133-7495-599B-B69B-95348B450DE7; **Taxon:** scientificName: Raviniapernix (Harris, 1780); order: Diptera; family: Sarcophagidae; genus: Ravinia; specificEpithet: pernix; scientificNameAuthorship: (Harris, 1780); **Location:** country: Pakistan; countryCode: PK; stateProvince: Balochistan; county: Ziatar District; municipality: Ziarat; decimalLatitude: 30.400139; decimalLongitude: 67.726806; geodeticDatum: WGS84; **Identification:** identifiedBy: Qaiser Khan; **Event:** eventDate: 2019-09-30
- **Type status:** Other material. **Occurrence:** recordedBy: Qaiser Khan; individualCount: 12; occurrenceID: 04E2787E-6153-578B-AA7E-671C7FA62453; **Taxon:** scientificName: Raviniapernix (Harris, 1780); order: Diptera; family: Sarcophagidae; genus: Ravinia; specificEpithet: pernix; scientificNameAuthorship: (Harris, 1780); **Location:** country: Pakistan; countryCode: PK; stateProvince: Balochistan; county: Ziatar District; municipality: Ziarat; decimalLatitude: 30.400139; decimalLongitude: 67.726806; geodeticDatum: WGS84; **Identification:** identifiedBy: Qaiser Khan; **Event:** eventDate: 2019-10-07
- **Type status:** Other material. **Occurrence:** recordedBy: Qaiser Khan; individualCount: 2; occurrenceID: A2D3AE72-FF9F-580E-939A-ECE3D4EE8BA0; **Taxon:** scientificName: Raviniapernix (Harris, 1780); order: Diptera; family: Sarcophagidae; genus: Ravinia; specificEpithet: pernix; scientificNameAuthorship: (Harris, 1780); **Location:** country: Pakistan; countryCode: PK; stateProvince: Balochistan; county: Ziatar District; municipality: Ziarat; decimalLatitude: 30.400139; decimalLongitude: 67.726806; geodeticDatum: WGS84; **Identification:** identifiedBy: Qaiser Khan; **Event:** eventDate: 2019-11-11
- **Type status:** Other material. **Occurrence:** recordedBy: Qaiser Khan; individualCount: 4; occurrenceID: 422ED40B-A1CC-57EF-9F35-ACDE36D1F5D8; **Taxon:** scientificName: Raviniapernix (Harris, 1780); order: Diptera; family: Sarcophagidae; genus: Ravinia; specificEpithet: pernix; scientificNameAuthorship: (Harris, 1780); **Location:** country: Pakistan; countryCode: PK; stateProvince: Balochistan; county: Ziatar District; municipality: Ziarat; decimalLatitude: 30.400139; decimalLongitude: 67.726806; geodeticDatum: WGS84; **Identification:** identifiedBy: Qaiser Khan; **Event:** eventDate: 2019-11-18

#### Distribution

Widely distributed in North America and Eurasia regions (Rafinejad et al. 2014, Nateghpour and Akbarzadeh 2017, Sharma et al. 2018). This species has been reported from Asian countries including Pakistan (Fatima and Yang 2022, Sugiama 1989).

#### Notes

**Figure 12.**
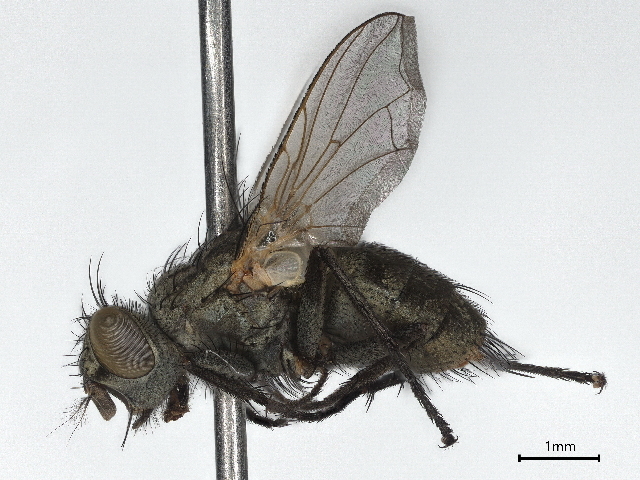
*Raviniapernix* (Thompson, 1869).

It is commonly referred to as the red-tailed flesh fly and is known to be a potential vector for disease due to its tendency to breed in carrion and faeces (Nateghpour and Akbarzadeh 2017). This species with the first documented record in the study area was observed from May to November (Fig. 12).

### Sarcophaga dux

Thomson, 1869

E38A0F29-3FB7-535C-91E9-BECEC2F2CCCB

OR685697

#### Materials

- **Type status:** Other material. **Occurrence:** recordedBy: Qaiser Khan; individualCount: 2; occurrenceID: E99372D4-C33D-5C67-876A-90C4C759F7CC; **Taxon:** scientificName: Sarcophagadux Thomson, 1869; order: Diptera; family: Sarcophagidae; genus: Sarcophaga; specificEpithet: dux; scientificNameAuthorship: Thomson, 1869; **Location:** country: Pakistan; countryCode: PK; stateProvince: Balochistan; county: Ziatar District; municipality: Ziarat; decimalLatitude: 30.400139; decimalLongitude: 67.726806; geodeticDatum: WGS84; **Identification:** identifiedBy: Qaiser Khan; **Event:** eventDate: 2019-05-27
- **Type status:** Other material. **Occurrence:** recordedBy: Qaiser Khan; individualCount: 1; occurrenceID: FF24595E-DFE5-5E00-9E4A-DBAC4F195616; **Taxon:** scientificName: Sarcophagadux Thomson, 1869; order: Diptera; family: Sarcophagidae; genus: Sarcophaga; specificEpithet: dux; scientificNameAuthorship: Thomson, 1869; **Location:** country: Pakistan; countryCode: PK; stateProvince: Balochistan; county: Ziatar District; municipality: Ziarat; decimalLatitude: 30.400139; decimalLongitude: 67.726806; geodeticDatum: WGS84; **Identification:** identifiedBy: Qaiser Khan; **Event:** eventDate: 2019-06-03
- **Type status:** Other material. **Occurrence:** recordedBy: Qaiser Khan; individualCount: 5; occurrenceID: EB678CDB-0B05-5412-9A7C-BDC2AA2E24B7; **Taxon:** scientificName: Sarcophagadux Thomson, 1869; order: Diptera; family: Sarcophagidae; genus: Sarcophaga; specificEpithet: dux; scientificNameAuthorship: Thomson, 1869; **Location:** country: Pakistan; countryCode: PK; stateProvince: Balochistan; county: Ziatar District; municipality: Ziarat; decimalLatitude: 30.400139; decimalLongitude: 67.726806; geodeticDatum: WGS84; **Identification:** identifiedBy: Qaiser Khan; **Event:** eventDate: 2019-06-17
- **Type status:** Other material. **Occurrence:** recordedBy: Qaiser Khan; individualCount: 1; occurrenceID: 7133E4F1-EDF3-5EF6-8F44-BDC71BBD5D69; **Taxon:** scientificName: Sarcophagadux Thomson, 1869; order: Diptera; family: Sarcophagidae; genus: Sarcophaga; specificEpithet: dux; scientificNameAuthorship: Thomson, 1869; **Location:** country: Pakistan; countryCode: PK; stateProvince: Balochistan; county: Ziatar District; municipality: Ziarat; decimalLatitude: 30.400139; decimalLongitude: 67.726806; geodeticDatum: WGS84; **Identification:** identifiedBy: Qaiser Khan; **Event:** eventDate: 2019-07-22
- **Type status:** Other material. **Occurrence:** recordedBy: Qaiser Khan; individualCount: 14; occurrenceID: 4504D2C3-5741-5749-BDE5-4044D283B0D4; **Taxon:** scientificName: Sarcophagadux Thomson, 1869; order: Diptera; family: Sarcophagidae; genus: Sarcophaga; specificEpithet: dux; scientificNameAuthorship: Thomson, 1869; **Location:** country: Pakistan; countryCode: PK; stateProvince: Balochistan; county: Ziatar District; municipality: Ziarat; decimalLatitude: 30.400139; decimalLongitude: 67.726806; geodeticDatum: WGS84; **Identification:** identifiedBy: Qaiser Khan; **Event:** eventDate: 2019-07-29
- **Type status:** Other material. **Occurrence:** recordedBy: Qaiser Khan; individualCount: 10; occurrenceID: DA109192-CEB6-5DB3-A83D-D782ADECB18A; **Taxon:** scientificName: Sarcophagadux Thomson, 1869; order: Diptera; family: Sarcophagidae; genus: Sarcophaga; specificEpithet: dux; scientificNameAuthorship: Thomson, 1869; **Location:** country: Pakistan; countryCode: PK; stateProvince: Balochistan; county: Ziatar District; municipality: Ziarat; decimalLatitude: 30.400139; decimalLongitude: 67.726806; geodeticDatum: WGS84; **Identification:** identifiedBy: Qaiser Khan; **Event:** eventDate: 2019-08-19
- **Type status:** Other material. **Occurrence:** recordedBy: Qaiser Khan; individualCount: 3; occurrenceID: 89068A9B-B68A-5627-8C8A-61D39DDFC269; **Taxon:** scientificName: Sarcophagadux Thomson, 1869; order: Diptera; family: Sarcophagidae; genus: Sarcophaga; specificEpithet: dux; scientificNameAuthorship: Thomson, 1869; **Location:** country: Pakistan; countryCode: PK; stateProvince: Balochistan; county: Ziatar District; municipality: Ziarat; decimalLatitude: 30.400139; decimalLongitude: 67.726806; geodeticDatum: WGS84; **Identification:** identifiedBy: Qaiser Khan; **Event:** eventDate: 2019-09-02
- **Type status:** Other material. **Occurrence:** recordedBy: Qaiser Khan; individualCount: 15; occurrenceID: 749AE668-F43D-5A4D-BABC-AFA0998CCEBC; **Taxon:** scientificName: Sarcophagadux Thomson, 1869; order: Diptera; family: Sarcophagidae; genus: Sarcophaga; specificEpithet: dux; scientificNameAuthorship: Thomson, 1869; **Location:** country: Pakistan; countryCode: PK; stateProvince: Balochistan; county: Ziatar District; municipality: Ziarat; decimalLatitude: 30.400139; decimalLongitude: 67.726806; geodeticDatum: WGS84; **Identification:** identifiedBy: Qaiser Khan; **Event:** eventDate: 2019-09-09
- **Type status:** Other material. **Occurrence:** recordedBy: Qaiser Khan; individualCount: 7; occurrenceID: 1FF2CCA3-8BBA-5D1A-8AE8-34312B7E79BE; **Taxon:** scientificName: Sarcophagadux Thomson, 1869; order: Diptera; family: Sarcophagidae; genus: Sarcophaga; specificEpithet: dux; scientificNameAuthorship: Thomson, 1869; **Location:** country: Pakistan; countryCode: PK; stateProvince: Balochistan; county: Ziatar District; municipality: Ziarat; decimalLatitude: 30.400139; decimalLongitude: 67.726806; geodeticDatum: WGS84; **Identification:** identifiedBy: Qaiser Khan; **Event:** eventDate: 2019-10-07
- **Type status:** Other material. **Occurrence:** recordedBy: Qaiser Khan; individualCount: 1; occurrenceID: 4581DF47-D934-52D7-A7BF-37B446B0B4EE; **Taxon:** scientificName: Sarcophagadux Thomson, 1869; order: Diptera; family: Sarcophagidae; genus: Sarcophaga; specificEpithet: dux; scientificNameAuthorship: Thomson, 1869; **Location:** country: Pakistan; countryCode: PK; stateProvince: Balochistan; county: Ziatar District; municipality: Ziarat; decimalLatitude: 30.400139; decimalLongitude: 67.726806; geodeticDatum: WGS84; **Identification:** identifiedBy: Qaiser Khan; **Event:** eventDate: 2019-10-14
- **Type status:** Other material. **Occurrence:** recordedBy: Qaiser Khan; individualCount: 3; occurrenceID: CE427440-A5AE-55AA-AB4D-CA6B629CF7DF; **Taxon:** scientificName: Sarcophagadux Thomson, 1869; order: Diptera; family: Sarcophagidae; genus: Sarcophaga; specificEpithet: dux; scientificNameAuthorship: Thomson, 1869; **Location:** country: Pakistan; countryCode: PK; stateProvince: Balochistan; county: Ziatar District; municipality: Ziarat; decimalLatitude: 30.400139; decimalLongitude: 67.726806; geodeticDatum: WGS84; **Identification:** identifiedBy: Qaiser Khan; **Event:** eventDate: 2019-11-18

#### Distribution

Cosmopolitan species (Byrd and Castner 2010, Chiewchanvit et al. 2017) also reported from Pakistan (Khoso et al. 2015).

#### Notes

**Figure 13.**
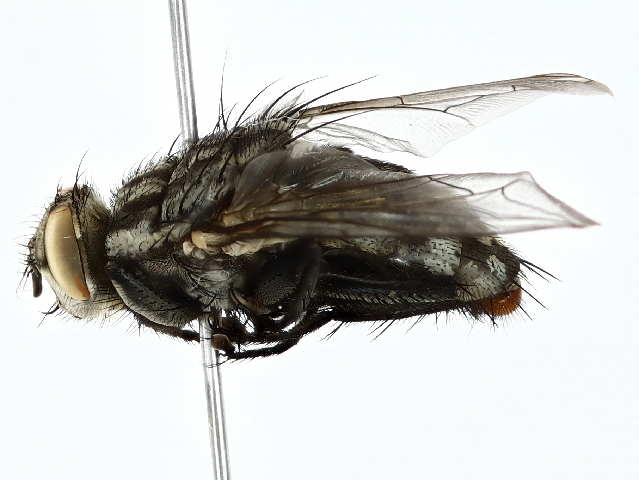
*Sarcophagadux* (Thompson, 1869).

This necrophagous species of forensic value for estimating minimum post-mortem interval (Zhang et al. 2020) has medical importance as a myiasis-producing agent. It has been documented for the first time in the study area. This species has been observed to exist from May to November (Fig. 13).

### Trupanea amoena

(Schiner, 1868)

CFAF6319-9C17-5C61-8E7B-889CFFBD997C

OR685698

#### Materials

- **Type status:** Other material. **Occurrence:** recordedBy: Qaiser Khan; individualCount: 2; occurrenceID: C0153CF7-F1D6-5445-B6B3-07BA56D6A4E4; **Taxon:** scientificName: Trupaneaamoena (Schiner, 1868); order: Diptera; family: Tephritidae; genus: Trupanea; specificEpithet: amoena; scientificNameAuthorship: (Schiner, 1868); **Location:** country: Pakistan; countryCode: PK; stateProvince: Balochistan; county: Ziatar District; municipality: Ziarat; decimalLatitude: 30.400139; decimalLongitude: 67.726806; geodeticDatum: WGS84; **Identification:** identifiedBy: Qaiser Khan; **Event:** eventDate: 2019-08-19
- **Type status:** Other material. **Occurrence:** recordedBy: Qaiser Khan; individualCount: 5; occurrenceID: B034FCBD-6108-53DA-8062-E34339835360; **Taxon:** scientificName: Trupaneaamoena (Schiner, 1868); order: Diptera; family: Tephritidae; genus: Trupanea; specificEpithet: amoena; scientificNameAuthorship: (Schiner, 1868); **Location:** country: Pakistan; countryCode: PK; stateProvince: Balochistan; county: Ziatar District; municipality: Ziarat; decimalLatitude: 30.400139; decimalLongitude: 67.726806; geodeticDatum: WGS84; **Identification:** identifiedBy: Qaiser Khan; **Event:** eventDate: 2019-09-16

#### Distribution

Species distributed from the Western Palearctic to the Oriental egion (Smit 2006, Merz 2008) and also reported from Pakistan (Punjab and Khyber Pakhtunkhwa Provinces) (Sarwar et al. 2014).

#### Notes

**Figure 14.**
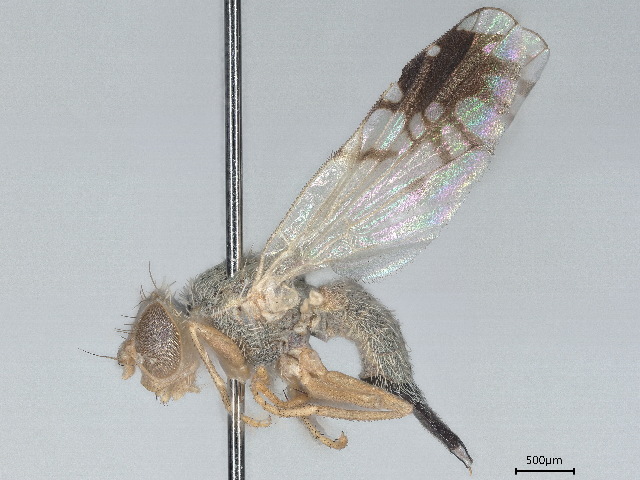
*Trupaneaamoena* (Schiner, 1868).

*Trupaneaamoena* is a serious agricultural pest known to infest cultivated and wild varieties of safflower as well as orchard plants (Sarwar et al. 2014). In the study area, this fly was recorded from August to September (Fig. 14).

### Wohlfahrtia bella

(Macquart, 1839)

E2DE56CD-AD3A-5B1A-AF3D-C9321E4BB609

OR685699

#### Materials

- **Type status:** Other material. **Occurrence:** recordedBy: Qaiser Khan; individualCount: 1; occurrenceID: 75D52789-D430-5AE1-B3CF-9F71ECB83BF3; **Taxon:** scientificName: Wohlfahrtiabella (Macquart, 1839); order: Diptera; family: Sarcophagidae; genus: Wohlfahrtia; specificEpithet: bella; scientificNameAuthorship: (Macquart, 1839); **Location:** country: Pakistan; countryCode: PK; stateProvince: Balochistan; county: Ziatar District; municipality: Ziarat; decimalLatitude: 30.400139; decimalLongitude: 67.726806; geodeticDatum: WGS84; **Identification:** identifiedBy: Qaiser Khan; **Event:** eventDate: 2019-04-22
- **Type status:** Other material. **Occurrence:** recordedBy: Qaiser Khan; individualCount: 1; occurrenceID: F9289AA5-4F77-59AB-9A31-519F7D574B51; **Taxon:** scientificName: Wohlfahrtiabella (Macquart, 1839); order: Diptera; family: Sarcophagidae; genus: Wohlfahrtia; specificEpithet: bella; scientificNameAuthorship: (Macquart, 1839); **Location:** country: Pakistan; countryCode: PK; stateProvince: Balochistan; county: Ziatar District; municipality: Ziarat; decimalLatitude: 30.400139; decimalLongitude: 67.726806; geodeticDatum: WGS84; **Identification:** identifiedBy: Qaiser Khan; **Event:** eventDate: 2019-05-27
- **Type status:** Other material. **Occurrence:** recordedBy: Qaiser Khan; individualCount: 3; occurrenceID: FB0F6700-591A-5C9A-87D8-861D8E26522E; **Taxon:** scientificName: Wohlfahrtiabella (Macquart, 1839); order: Diptera; family: Sarcophagidae; genus: Wohlfahrtia; specificEpithet: bella; scientificNameAuthorship: (Macquart, 1839); **Location:** country: Pakistan; countryCode: PK; stateProvince: Balochistan; county: Ziatar District; municipality: Ziarat; decimalLatitude: 30.400139; decimalLongitude: 67.726806; geodeticDatum: WGS84; **Identification:** identifiedBy: Qaiser Khan; **Event:** eventDate: 2019-06-10
- **Type status:** Other material. **Occurrence:** recordedBy: Qaiser Khan; individualCount: 1; occurrenceID: A1B5F324-0A68-515E-B788-929A02E1A659; **Taxon:** scientificName: Wohlfahrtiabella (Macquart, 1839); order: Diptera; family: Sarcophagidae; genus: Wohlfahrtia; specificEpithet: bella; scientificNameAuthorship: (Macquart, 1839); **Location:** country: Pakistan; countryCode: PK; stateProvince: Balochistan; county: Ziatar District; municipality: Ziarat; decimalLatitude: 30.400139; decimalLongitude: 67.726806; geodeticDatum: WGS84; **Identification:** identifiedBy: Qaiser Khan; **Event:** eventDate: 2019-06-24
- **Type status:** Other material. **Occurrence:** recordedBy: Qaiser Khan; individualCount: 8; occurrenceID: 8D812F1D-3468-580E-9CAA-BFD511AF8E52; **Taxon:** scientificName: Wohlfahrtiabella (Macquart, 1839); order: Diptera; family: Sarcophagidae; genus: Wohlfahrtia; specificEpithet: bella; scientificNameAuthorship: (Macquart, 1839); **Location:** country: Pakistan; countryCode: PK; stateProvince: Balochistan; county: Ziatar District; municipality: Ziarat; decimalLatitude: 30.400139; decimalLongitude: 67.726806; geodeticDatum: WGS84; **Identification:** identifiedBy: Qaiser Khan; **Event:** eventDate: 2019-07-29
- **Type status:** Other material. **Occurrence:** recordedBy: Qaiser Khan; individualCount: 21; occurrenceID: 7EEE108E-1335-5BFD-8F8D-79636155F946; **Taxon:** scientificName: Wohlfahrtiabella (Macquart, 1839); order: Diptera; family: Sarcophagidae; genus: Wohlfahrtia; specificEpithet: bella; scientificNameAuthorship: (Macquart, 1839); **Location:** country: Pakistan; countryCode: PK; stateProvince: Balochistan; county: Ziatar District; municipality: Ziarat; decimalLatitude: 30.400139; decimalLongitude: 67.726806; geodeticDatum: WGS84; **Identification:** identifiedBy: Qaiser Khan; **Event:** eventDate: 2019-08-19
- **Type status:** Other material. **Occurrence:** recordedBy: Qaiser Khan; individualCount: 21; occurrenceID: 923E29C2-A5B1-50F0-A2C5-5AA101F6596E; **Taxon:** scientificName: Wohlfahrtiabella (Macquart, 1839); order: Diptera; family: Sarcophagidae; genus: Wohlfahrtia; specificEpithet: bella; scientificNameAuthorship: (Macquart, 1839); **Location:** country: Pakistan; countryCode: PK; stateProvince: Balochistan; county: Ziatar District; municipality: Ziarat; decimalLatitude: 30.400139; decimalLongitude: 67.726806; geodeticDatum: WGS84; **Identification:** identifiedBy: Qaiser Khan; **Event:** eventDate: 2019-09-09
- **Type status:** Other material. **Occurrence:** recordedBy: Qaiser Khan; individualCount: 2; occurrenceID: 0CA593DF-B1E9-5C0C-A13A-F89A4325F187; **Taxon:** scientificName: Wohlfahrtiabella (Macquart, 1839); order: Diptera; family: Sarcophagidae; genus: Wohlfahrtia; specificEpithet: bella; scientificNameAuthorship: (Macquart, 1839); **Location:** country: Pakistan; countryCode: PK; stateProvince: Balochistan; county: Ziatar District; municipality: Ziarat; decimalLatitude: 30.400139; decimalLongitude: 67.726806; geodeticDatum: WGS84; **Identification:** identifiedBy: Qaiser Khan; **Event:** eventDate: 2019-09-16
- **Type status:** Other material. **Occurrence:** recordedBy: Qaiser Khan; individualCount: 11; occurrenceID: F29D05B6-592C-5CE4-9B0A-BCC74BFE325B; **Taxon:** scientificName: Wohlfahrtiabella (Macquart, 1839); order: Diptera; family: Sarcophagidae; genus: Wohlfahrtia; specificEpithet: bella; scientificNameAuthorship: (Macquart, 1839); **Location:** country: Pakistan; countryCode: PK; stateProvince: Balochistan; county: Ziatar District; municipality: Ziarat; decimalLatitude: 30.400139; decimalLongitude: 67.726806; geodeticDatum: WGS84; **Identification:** identifiedBy: Qaiser Khan; **Event:** eventDate: 2019-10-07
- **Type status:** Other material. **Occurrence:** recordedBy: Qaiser Khan; individualCount: 7; occurrenceID: BB9CDB29-A8FD-5FFB-8FA4-A9A311D4E4D4; **Taxon:** scientificName: Wohlfahrtiabella (Macquart, 1839); order: Diptera; family: Sarcophagidae; genus: Wohlfahrtia; specificEpithet: bella; scientificNameAuthorship: (Macquart, 1839); **Location:** country: Pakistan; countryCode: PK; stateProvince: Balochistan; county: Ziatar District; municipality: Ziarat; decimalLatitude: 30.400139; decimalLongitude: 67.726806; geodeticDatum: WGS84; **Identification:** identifiedBy: Qaiser Khan; **Event:** eventDate: 2019-11-18

#### Distribution

Cosmopolitan species (Pekbey and Hayat 2013), already recorded in Pakistan (Hall et al. 2009).

#### Notes

**Figure 15.**
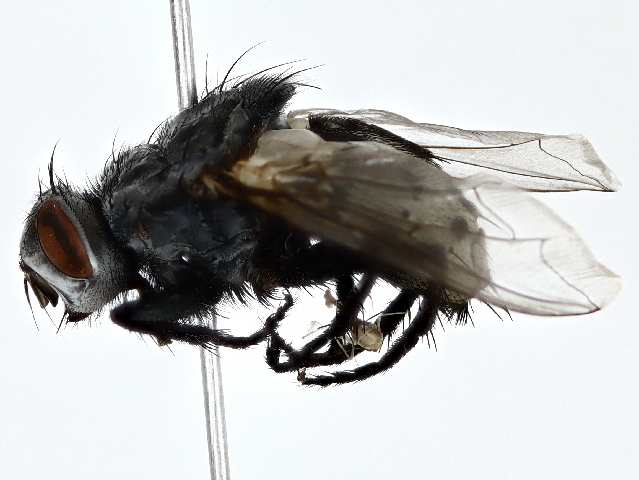
*Wohlfahrtiabella* (Linnaeus, 1758).

It holds medical importance and serves as a useful forensic tool for determining the minimum post-mortem interval (Zhang et al. 2021, Wang et al. 2011, Mingfu et al. 2006). Its presence was observed during April and November in the study area (Fig. 15).

## Discussion

DNA barcoding has been widely used as a taxonomic tool for species identification and cryptic species discovery. Previous studies have shown the great potential of DNA barcoding for biotic assessments in various contexts, including Malaise trap surveys (Chimeno et al. 2022, Moiz and Naqvi 1968, deWaard et al. 2019b, Geiger et al. 2016). This trap is particularly effective for capturing low-flying insects, especially within the Diptera and Hymenoptera categories (deWaard et al. 2019b). The utilization of both DNA barcoding and Malaise traps effectively facilitates species surveys on a larger scale when contrasted with traditional morphological methods within a shorter timeframe and at reduced costs (Ji et al. 2013, Hajibabaei et al. 2011). This research was conducted with the goal of developing a library of genetic barcodes for one of the important Pakistan's conservation area in Pakistan. However, the challenge of developing comprehensive reference libraries to identify a given taxonomic group is one of the primary difficulties in DNA barcoding (Virgilio et al. 2012). Amongst insect species, Dipteran are the largest and the most difficult to differentiate amongst other insects. Despite this distinction, they have received less attention compared to insect orders with lower species diversity (for example, Lepidoptera, Coleoptera and including those species found in fresh water habitats). These orders demand only basic taxonomic skills for differentiation (Morinière et al. 2019).

We have identified 14 species of Diptera through DNA barcode sequencings. Syrphidae was the most represented with four genera and five species, followed by Sarcophagidae with three genera and three species. Muscidae and Anthomyiidae were each represented by one genus and three species. The remaining three families namely Polleniidae, Drosophilidae and Tephritidae, were each represented by one genus and two species. Amongst these, the genera within Syrphidae and *Lucilia* displayed maximum p-distances of 3.5%, 4.49% and 4.04%, respectively, surpassing the p-distances of their nearest neighbours. DNA barcodes were generated for only 1427 (58.69%) of these identified Dipteran specimens. This could be attributed to DNA contamination or damage during the extraction process. In an insect biodiversity surveillance effort in Pakistan, Ashfaq et al. (2022) generated DNA barcodes for 60,273 specimens, leaving 17% of the specimens without DNA barcodes. Similarly, other studies by Geiger et al. (2016) and Pentinsaari et al. (2020) reported discrepancies in the sequences derived from various insect taxa. In our study, of the 27 barcoded specimens, 12 had complete matches with the NCBI database, while six revealed a 99% to 99.85% match. One species showed a 98.33% match and four species had matches ranging from 90.0% to 97.0% and the remaining species displayed a nucleotide identity of over 84.0% with the NCBI database.

Through a comparison of species diagnoses, based on morphology and molecular characteristics, we have produced a first, despite obviously largely incomplete, inventory of Diptera within the Juniper forest of the Ziarat region. This area has been largely unexplored in terms of its insect biodiversity. Notably, our study highlights the first occurrence of *Goniaoranta* in Pakistan, previously first documented in Russia by Ziegler and Shima (1996). This new record holds significance due to the species role as a parasitic predator of lepidopterous larvae, offering valuable control against insect pests, such as the destructive cutworm outbreaks in the grain-growing regions of Western Canada. Additionally, the other species documented in our research mark the first records for Balochistan and, thus, extending species distribution in Pakistan (Selivon et al. 2022, Clarke et al. 2005, Adler et al. 2020). These surveys will also help to verify whether these genera possess distinct localization within diverse biomes across other protected regions.

In this research, Intraspecific polymorphism was observed between only *A.soccata* and *A.varia*, showing that all the other morphologically distinct species have acquired distinctive haplotypes. The evaluation of intraspecific and interspecific differences in species is typically observed when multiple taxa with larger specimens are involved in a study. However, in the case of the study conducted by Savage et al. (2004),where it was unique results because it included only a single specimen from each taxon for analysis. The presence of these unique species underscores the importance of preserving their natural habitats. This leads us to suspect that *A.soccata* and *A.varia* may be part of the same polymorphic species. To determine whether this is the case,it need to be measured the genetic distance between these two species by using additional markers that can distinguish between closely-related species (Whitworth et al. 2007). Further, this investigation holds the potential to unveil a richer array of cryptic species within the fauna of this ecosystem. It is worth mentioning about the absence of prior investigations within protected areas such as *Juniperous*. Subsequent faunistic surveys are important to gauge the abundance and precise distribution patterns of the identified Diptera genera.

Ssymank et al. (2008) and Orford et al. (2015) have reported that many species of mayflies contribute to ecological services, such as pollination. For instance, in our study of migratory species, i.e., *Eristalistenax* acts as a pollinator and was more abundant in summer, while *Eupeodescorollae* also contributes to pollination and can be seen during the same season. A third species, *Goniaornata* was present from January till the end of the year, proving its relevance in pollinating flowers. Schenk et al. (2018) has suggested that changes in phenology might lead to a mismatch between pollinators and flowers, as different systems have various phenological responses. Our findings corroborate the anticipation that species density tends to be reduced during the dry season, except for *Goniaornata*. The majority of species occurrences were noted starting from April. This trend aligns with observations from a separate study, where the peak presence was recorded even earlier in March-April (Thompson and Rotheray 1998). Our findings potentially indicate that the pollinators in our study area might not be significantly influenced by climatic conditions. Nevertheless, this pattern diverged in the case of *Atherigonavaria* and *Polleniarudis*. These species displayed brief appearances throughout the sampling year. Within our study area, the dry season exerts considerable environmental stress on most insects, characterized by temperatures and humidity levels below their optimal thresholds. It remains plausible that the decline in population of these species during this period stems from an overall reduction in available resources. It has been observed that insects can demonstrate fluctuations in species density under distinct environmental circumstances suggesting that these variations in density (and diversity) arise from a combination of environmental pressures and competitive interactions (Chown and Terblanche 2006, Ezeakacha and Yee 2019). Consequently, we propose the importance of constructing a phenology-based statistical model for investigating life-table parameters across different temperature regimes, as highlighted by Valadao et al. (2019).

## Supplementary Material

XML Treatment for Adia cinerella

XML Treatment for Atherigona soccata

XML Treatment for Atherigona varia

XML Treatment for Chironomus dorsalis

XML Treatment for Eupeodes corollae

XML Treatment for Eristalis tenax

XML Treatment for Gonia ornata

XML Treatment for Lucilia sericata

XML Treatment for Paragus quadrifasciatus

XML Treatment for Pollenia rudis

XML Treatment for Ravinia pernix

XML Treatment for Sarcophaga dux

XML Treatment for Trupanea amoena

XML Treatment for Wohlfahrtia bella
